# Supplementary material for: Iridium-Catalyzed and pH-Dependent Reductions of Nitroalkenes to Ketones
Source: Molecules. 2022 Nov 13;27(22):7822. doi: 10.3390/molecules27227822 (PMC9696932; doi:10.3390/molecules27227822)

Supporting Information

Iridium-Catalyzed and pH-Dependent Reductions of Nitroalkenes to Ketones

Tingting Wang,† Changmeng Liu,† Dong Xu, Jiaxi Xu, Zhanhui Yang*

Department of Organic Chemistry, College of Chemistry, Beijing University of Chemical Technology, Beijing 100029, People's Republic of China

†These authors contributed equally.
*Correspondence: zhyang@mail.buct.edu.cn

Table of Contents

1. Methods for calculating the product distribution..1

2. Gram-scale synthesis..2

3. Copies of NMR Spectra 2

3.1 ^{1}H and ^{13}C Spectra of products...2

3.2 ^{1}H NMR for Reaction Mixtures in Table 1...18

1. Methods for calculating the product distribution

All the α-methyl-β-(4-substituted-phenyl)nitroethylenes, the nitroalkanes, oximes, and ketones products are known compounds. Thus, we used the ^{1}H NMR spectra to analyze the crude reaction mixtures in the optimization of the conditions. We take the ^{1}H NMR of the crude reaction mixture in Table 1, entry 3 (Figure S1) as an example. The data were summarized in Table S1.

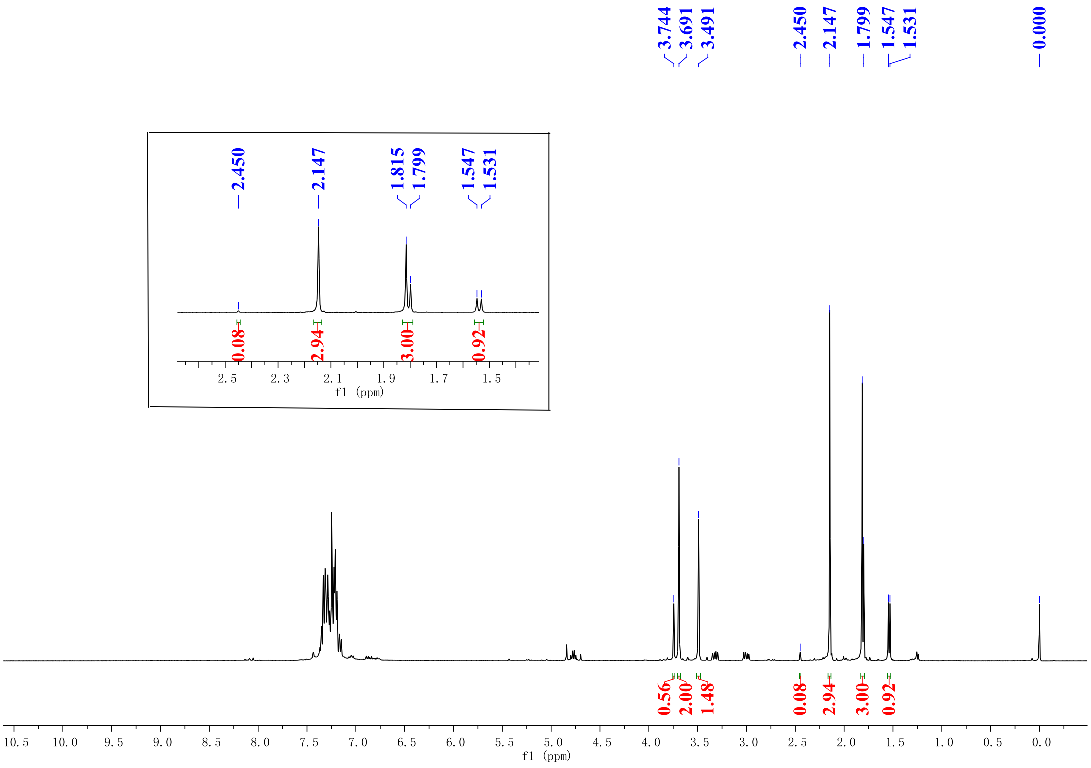

Figure S1. The ^{1}H NMR of the crude reaction mixture in Table 1, entry 3

This mixture contains nitroalkene (**1a**), nitroalkane (**2a**), *Z*- and *E*-oxime (**3a**), and ketone (**4a**). All these four kinds of compounds contain electronically different methyl groups, which were well discriminated by the ^{1}H NMR.

Table S1. Methods for calculating the product distribution and yield.

Compound	**1a**	**2a**	**3a**	**4a**
δ (Me)	2.45 (s)	1.54 (d, J = 6.6 Hz, 1H).	1.82 (s), 1.80 (s)	2.15 (s)
Integral	0.08	0.92	3.00	2.94
Molar ratio (%)	1	13	43	43

Therefore, the conversion of **1a** was 99%; the yield of **2a** is 13%; the yield of **3a** is 43%; the yield of **4a** is 43%.

2. Gram-scale synthesis

A 250-mL round bottom flask was charged with (2-nitroprop-1-en-1-yl)benzene (**1a**) (1.50 g, 9.2 mmol), EtOH (36.8 mL), **C3** catalyst (2.1 mg, 0.00368 mmol), deionized water (36.8 mL), HCO_2H (2.84 mL), and H_2SO_4 (3.68 mol/L, 0.92 mL). The mixture was stirred at 80 ºC for 3 h. Then HCl (36.8 mL) was added and the mixture was stirred for another 1 h. After cooling to room temperature, diluting with water (74 mL), extracting with ethyl acetate (100 mL x 3), the organic phase was washed with saturated sodium bicarbonate and dried over anhydrous sodium sulfate. The organic phase was evaporated under reduced pressure and the residue was purified by flash column chromatography to afford 1-phenylpropan-2-one (**4a**) as a yellowish oil (0.86 g, 70%).

3. Copies of NMR Spectra

3.1 ^{1}H and ^{13}C Spectra of products

^{1}H NMR spectrum (400 MHz, $CDCl_3$) of 1-phenylpropan-2-one (**4a**)

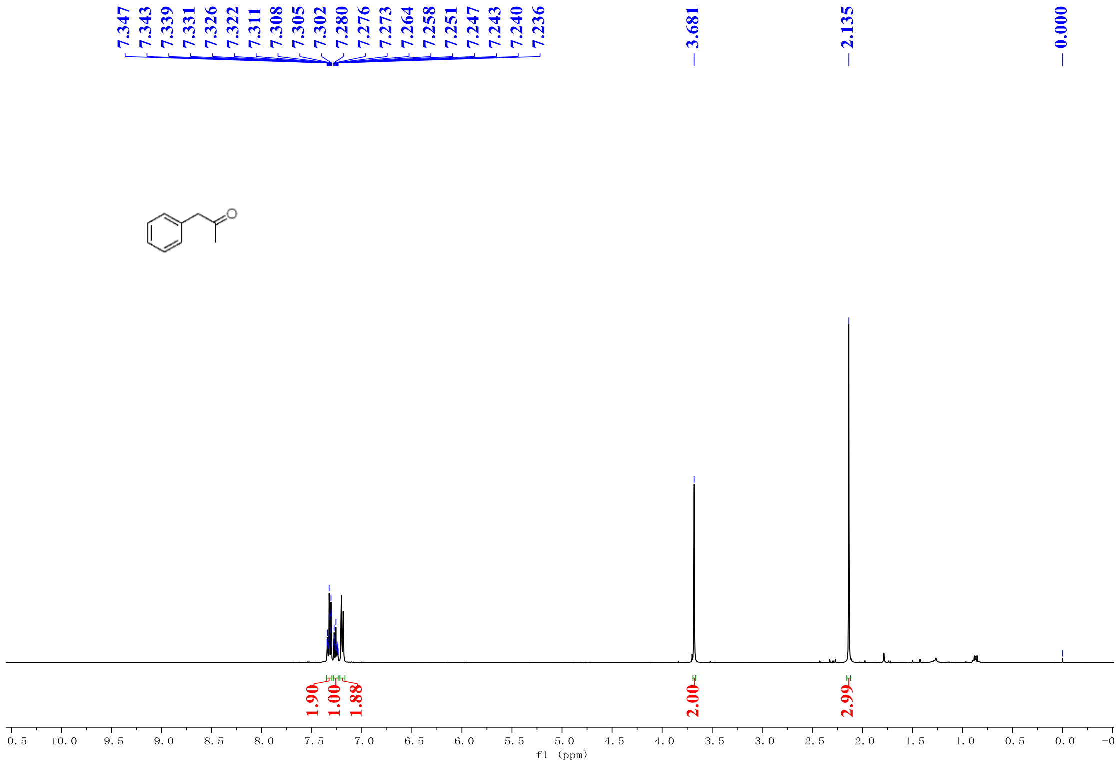

^{13}C NMR spectrum (101 MHz, $CDCl_3$) of 1-Phenylpropan-2-one (**4a**)

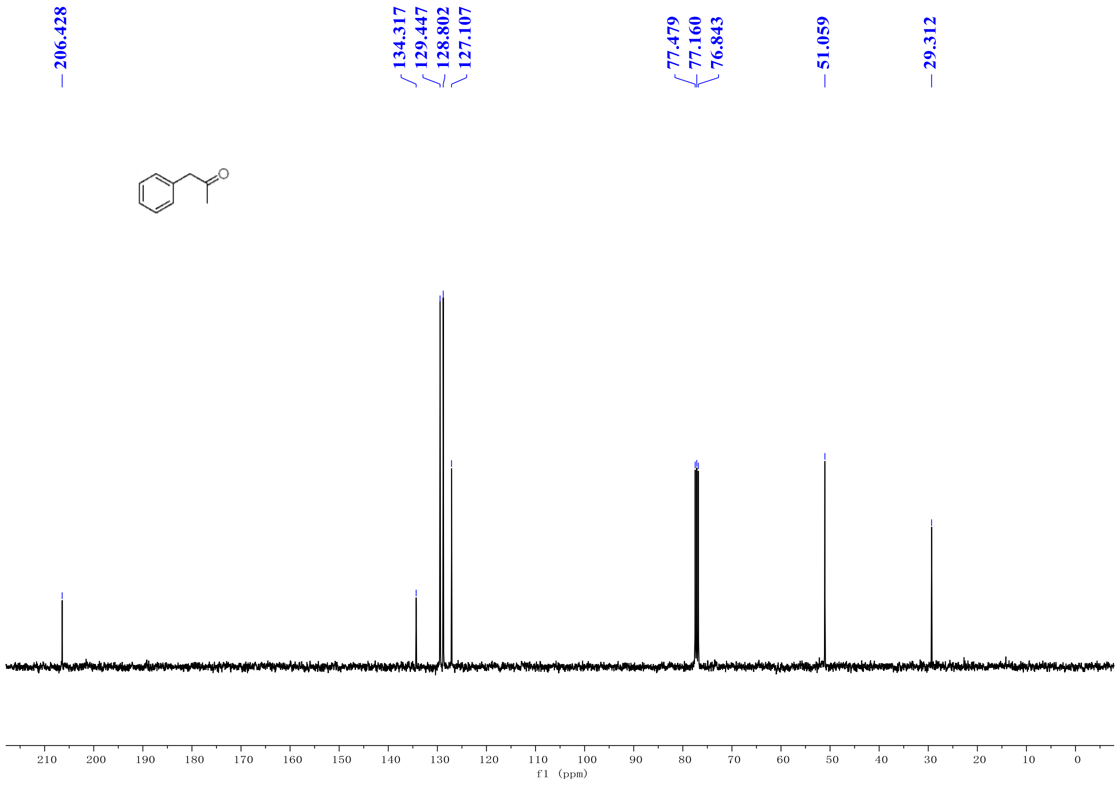

^1H NMR spectrum (400 MHz, $CDCl_3$) of 1-(4-methoxyphenyl)propan-2-one (**4b**)

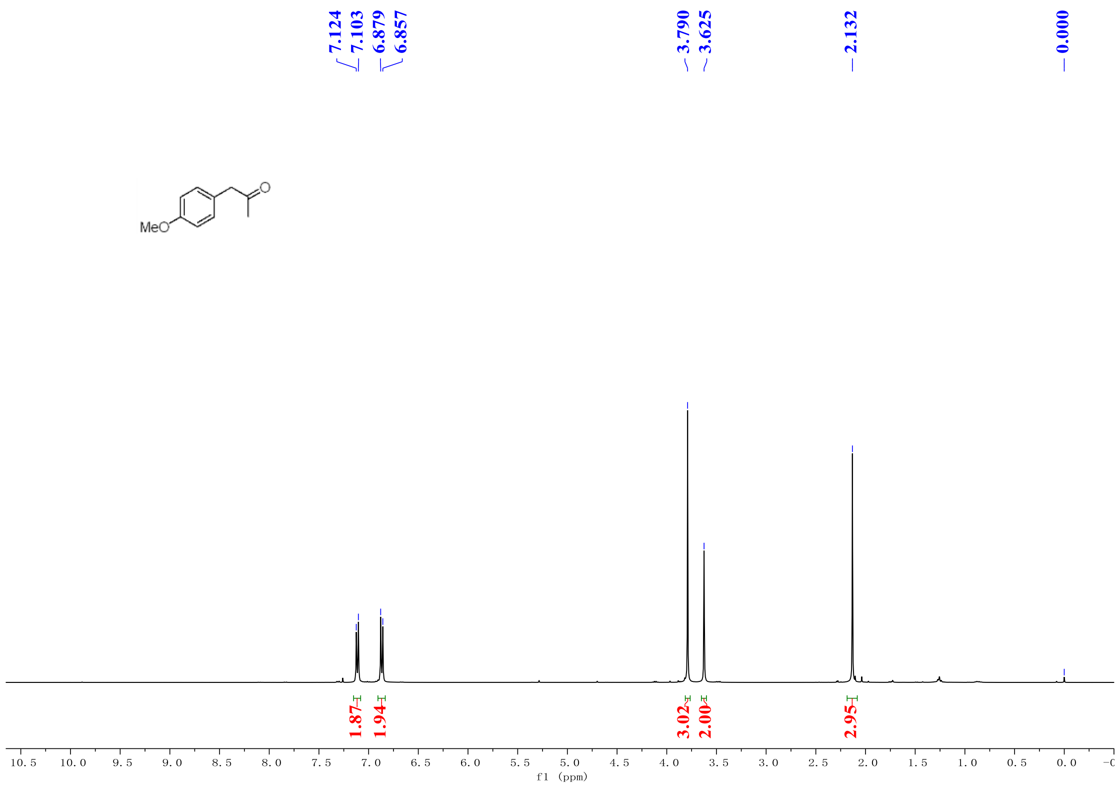

^{13}C NMR spectrum (101 MHz, $CDCl_3$) of 1-(4-methoxyphenyl)propan-2-one (**4b**)

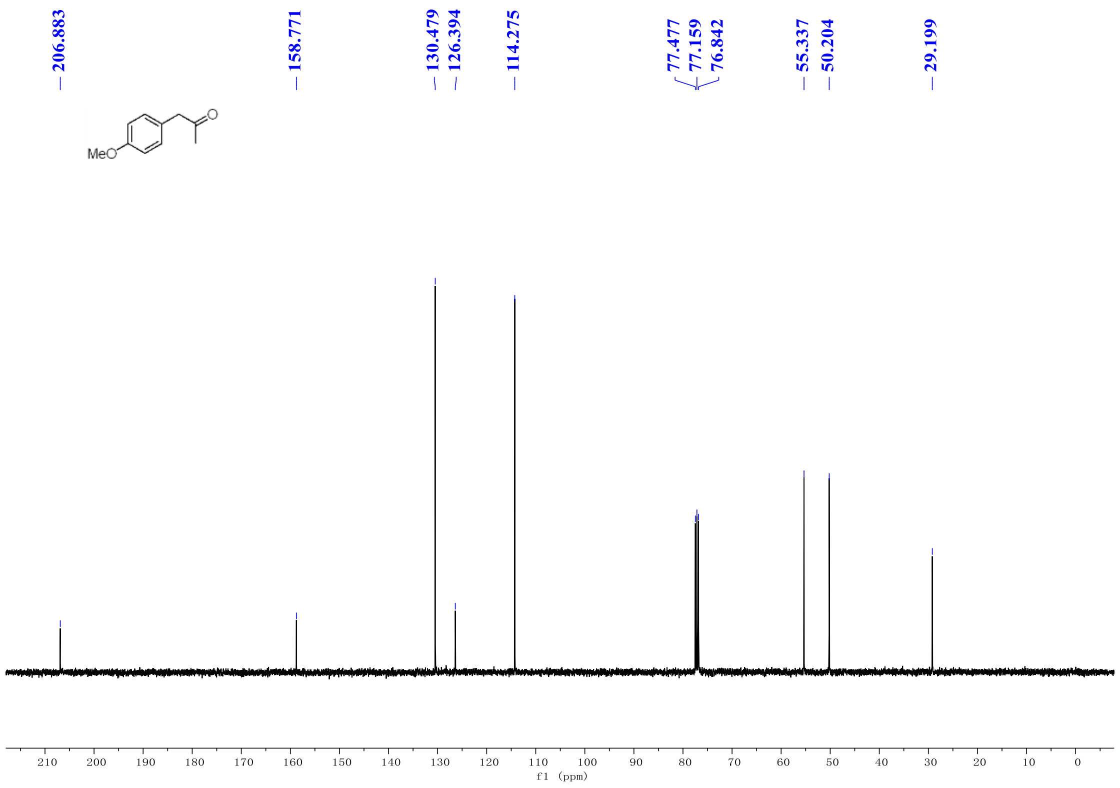

^{1}H NMR spectrum (400 MHz, $CDCl_3$) of 1-(4-(methylthio)phenyl)propan-2-one (**4c**)

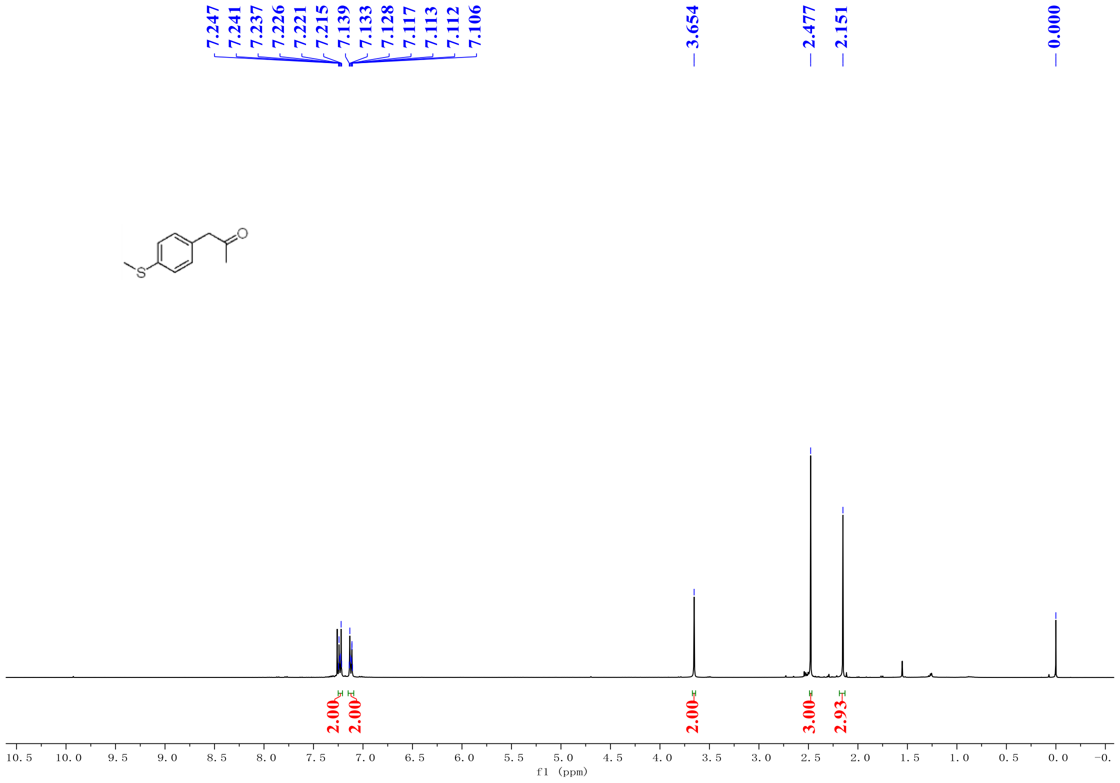

^{13}C NMR spectrum (101 MHz, $CDCl_3$) of 1-(4-(methylthio)phenyl)propan-2-one (**4c**)

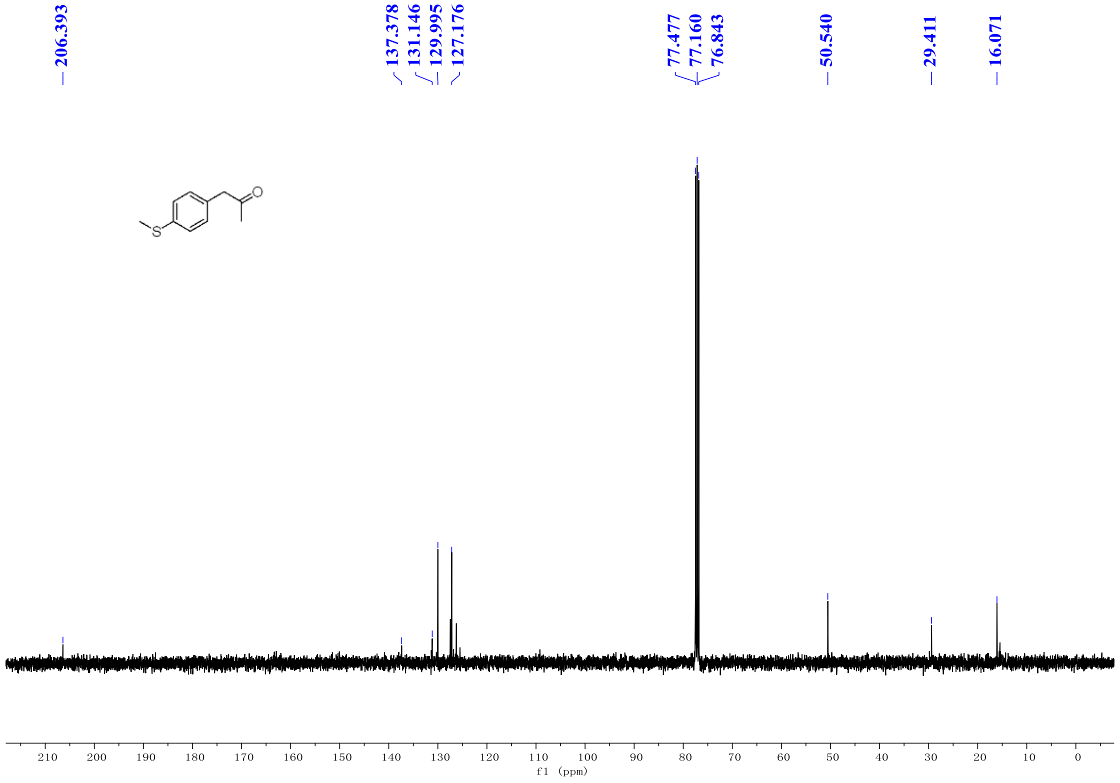

^{1}H NMR spectrum (400 MHz, $CDCl_3$) of 1-(*p*-tolyl)propan-2-one (**4d**)

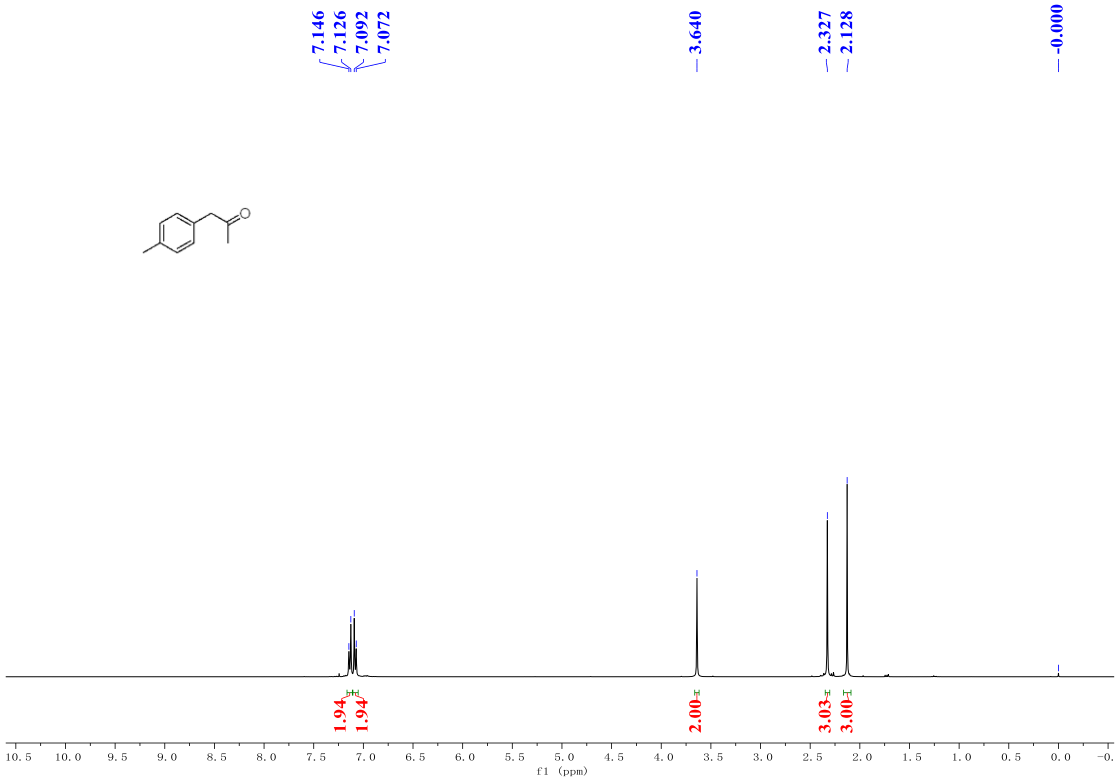

^{13}C NMR spectrum (101 MHz, $CDCl_3$) of 1-(*p*-tolyl)propan-2-one (**4d**)

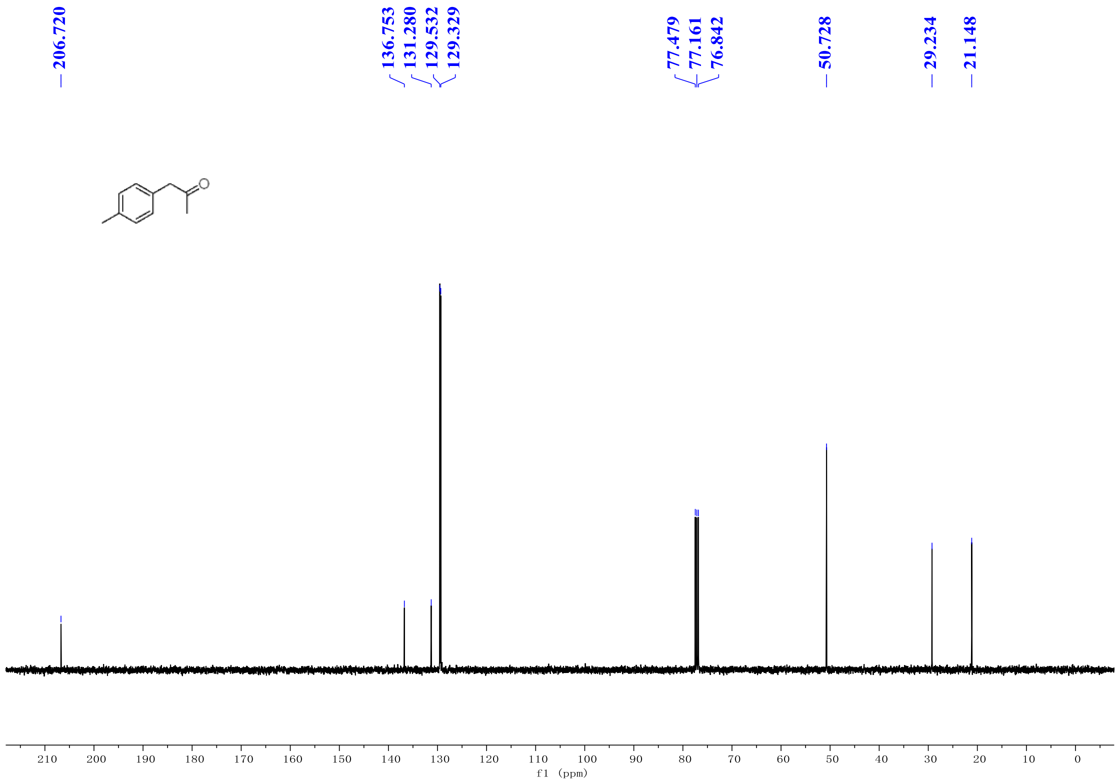

^{1}H NMR spectrum (400 MHz, $CDCl_3$) of 1-(4-fluorophenyl)propan-2-one (**4e**)

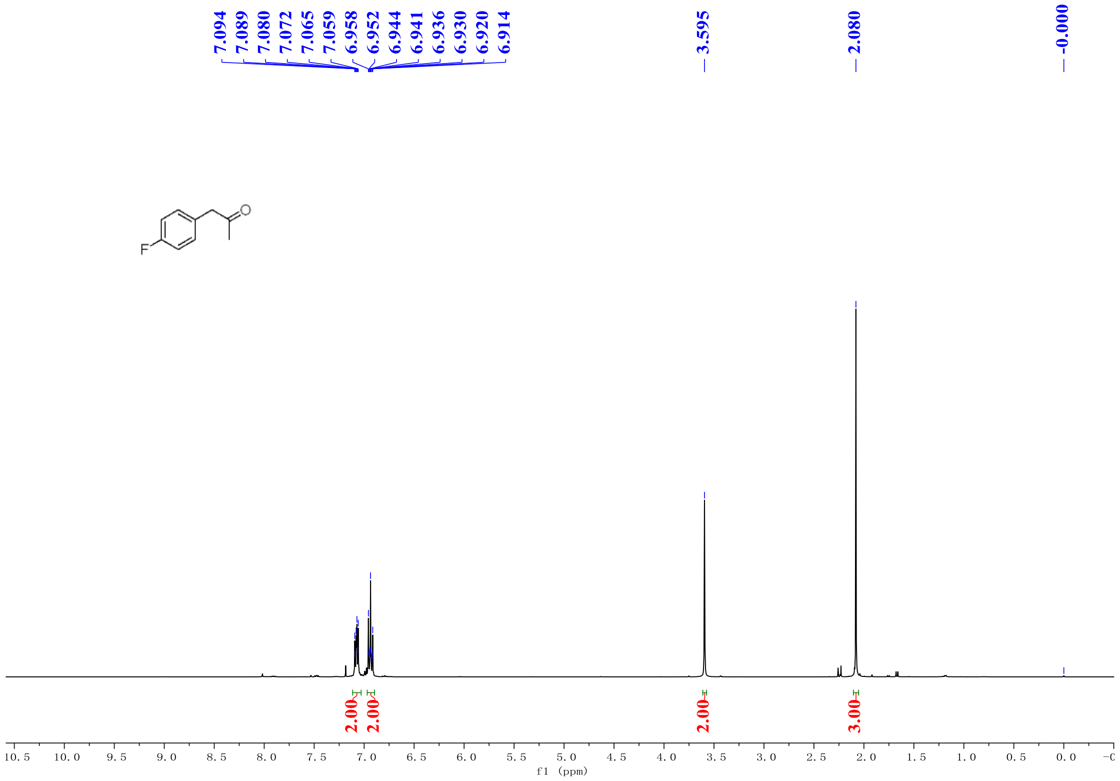

^{13}C NMR spectrum (101 MHz, $CDCl_3$) of 1-(4-fluorophenyl)propan-2-one (**4e**)

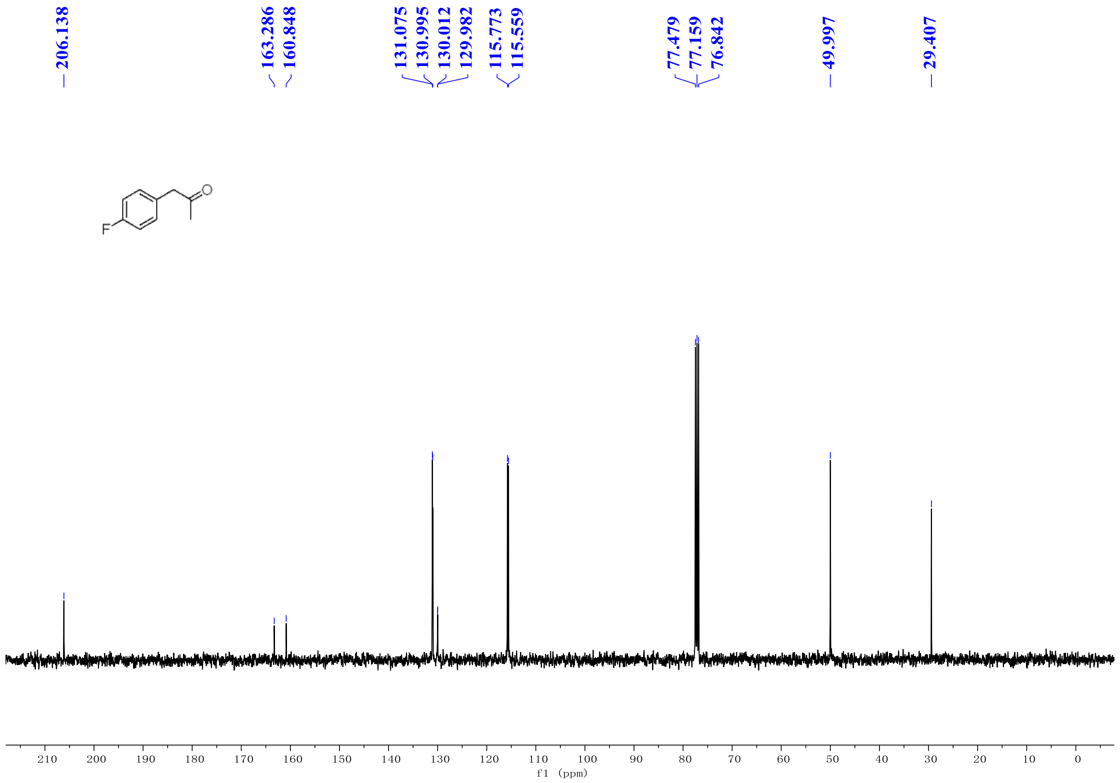

^{19}F NMR spectrum (377 MHz, $CDCl_3$) of 1-(4-fluorophenyl)propan-2-one (**4e**)

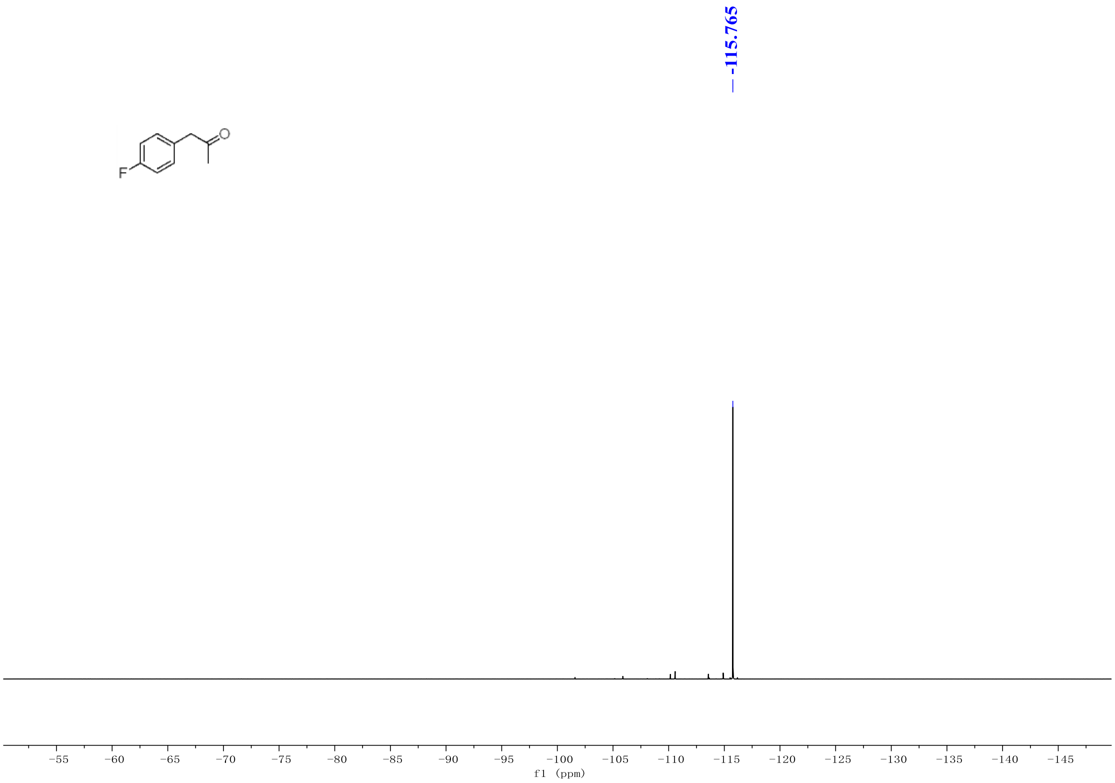

^{1}H NMR spectrum (400 MHz, $CDCl_3$) of1-(4-chlorophenyl)propan-2-one (**4f**)

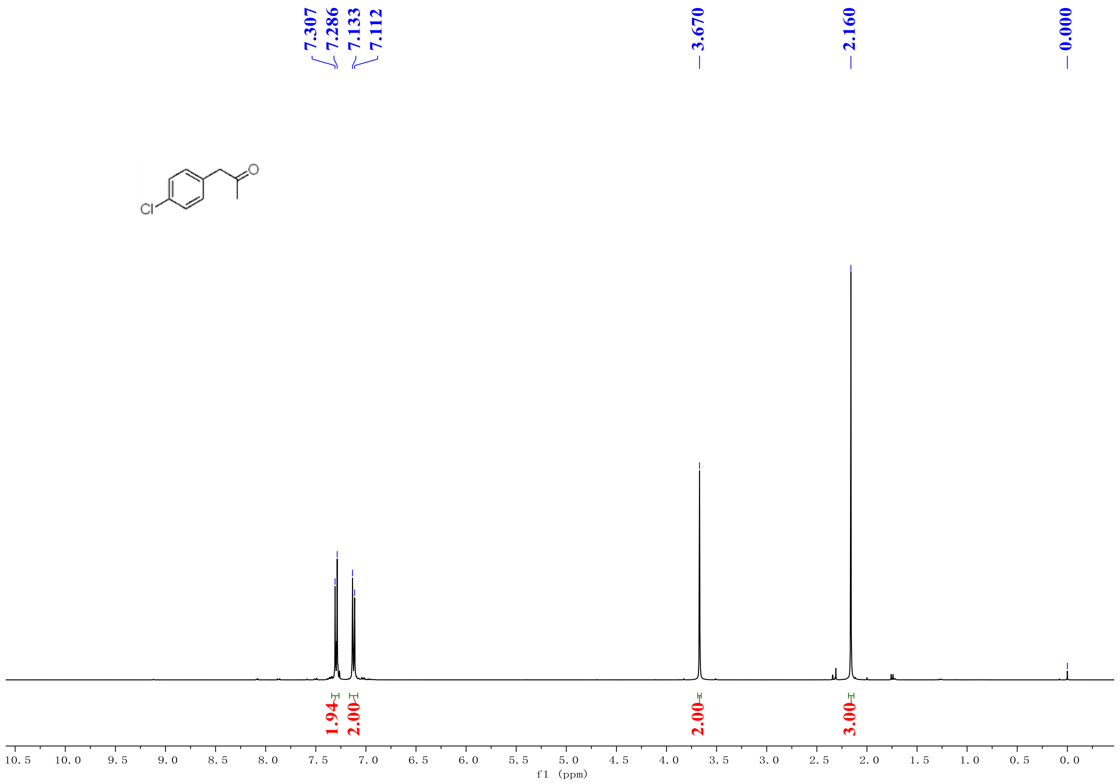

^{13}C NMR spectrum (101 MHz, $CDCl_3$) of 1-(4-chlorophenyl)propan-2-one (**4f**)

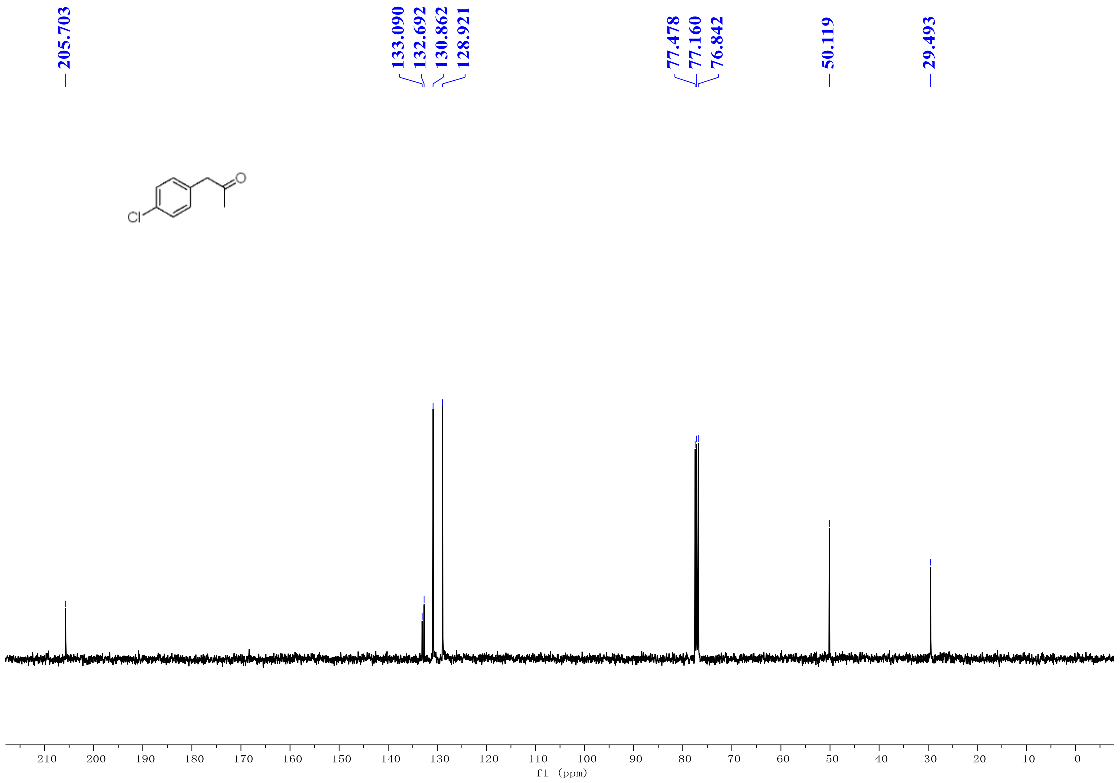

^{1}H NMR spectrum (400 MHz, $CDCl_3$) of 1-(4-bromophenyl)propan-2-one (**4g**)

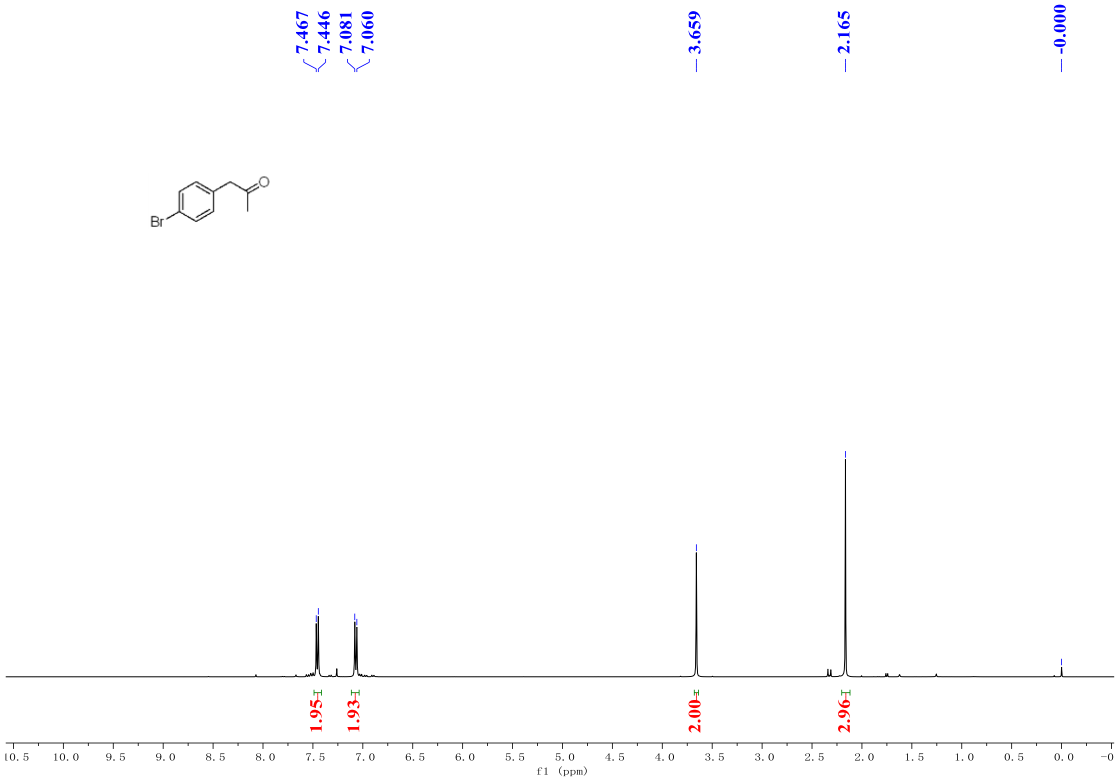

^{13}C NMR spectrum (101 MHz, $CDCl_3$) of 1-(4-bromophenyl)propan-2-one (**4g**)

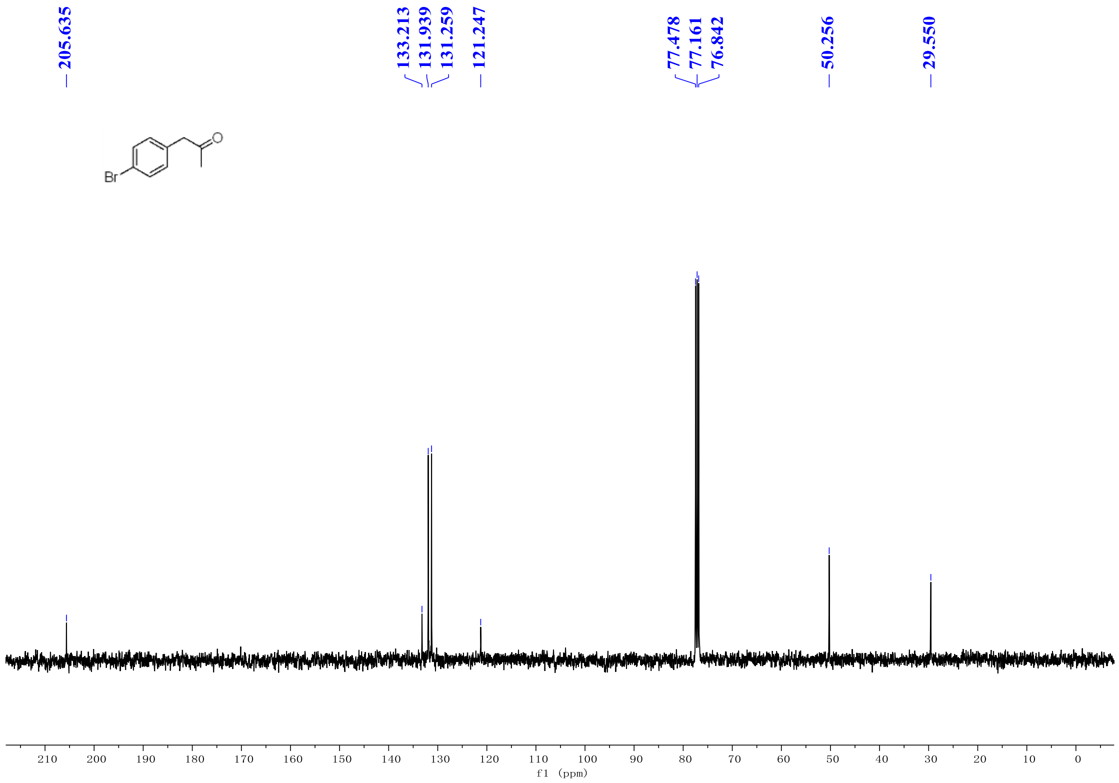

^{1}H NMR spectrum (400 MHz, $CDCl_3$) of 1-(4-(trifluoromethyl)phenyl)propan-2-one (**4h**)

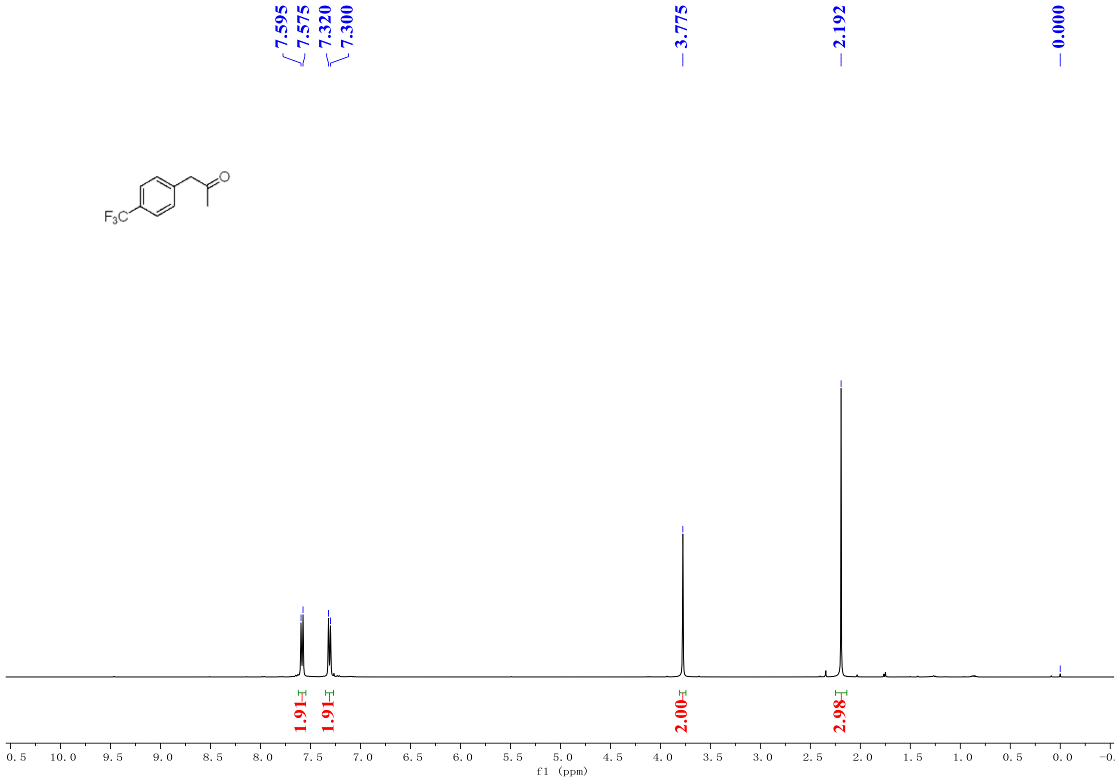

^{13}C NMR spectrum (101 MHz, $CDCl_3$) of 1-(4-(trifluoromethyl)phenyl)propan-2-one

(**4h**)

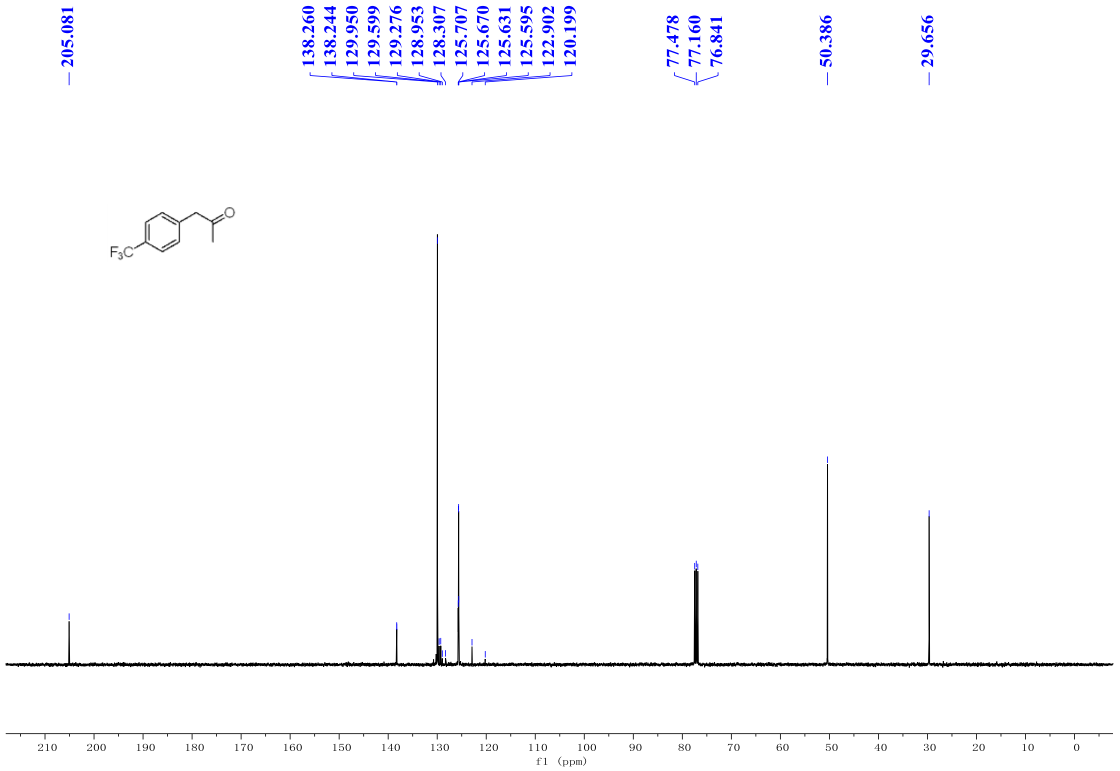

^{19}F NMR spectrum (377 MHz, $CDCl_3$) of 1-(4-(trifluoromethyl)phenyl)propan-2-one (**4h**)

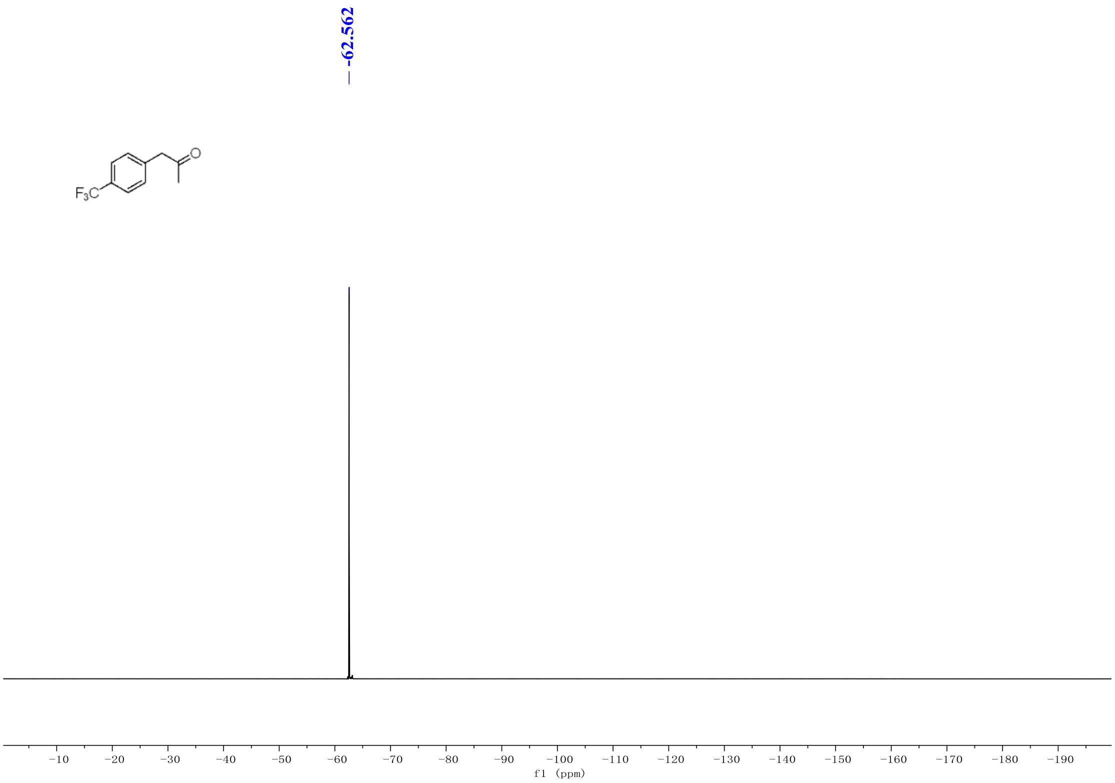

^1H NMR spectrum (400 MHz, $CDCl_3$) of 4-(2-oxopropyl)benzonitrile (**4i**)

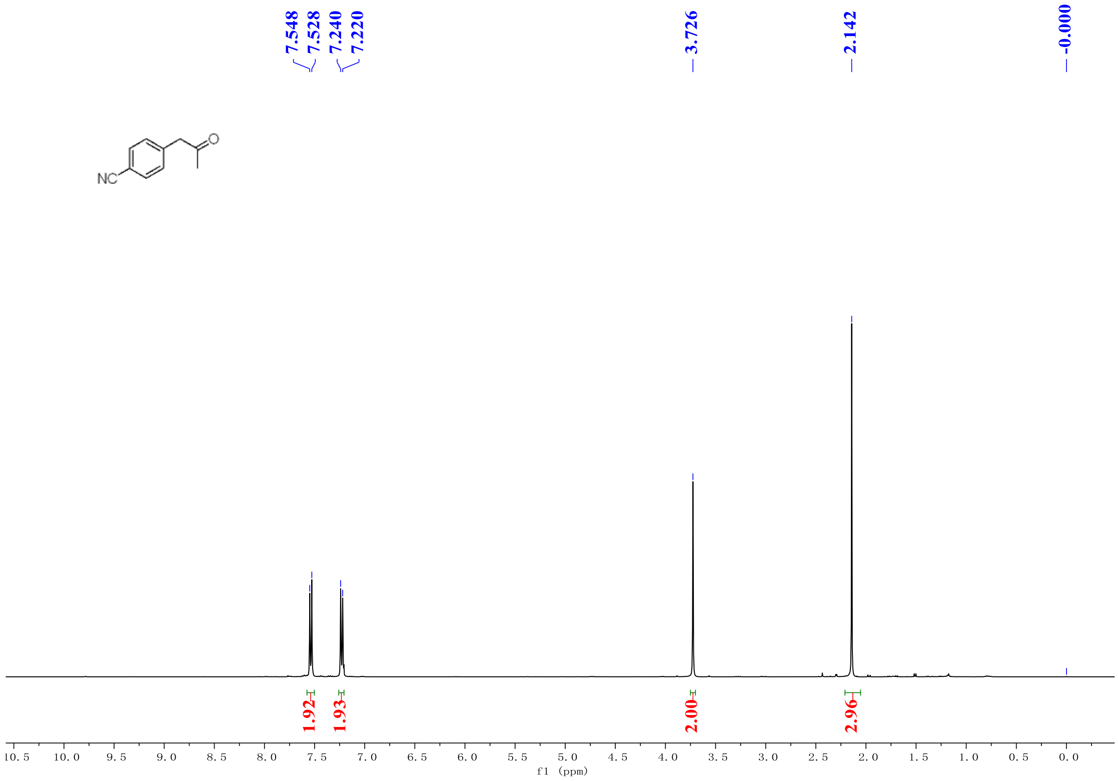

^{13}C NMR spectrum (101 MHz, $CDCl_3$) of compound 4-(2-oxopropyl)benzonitrile (**4i**)

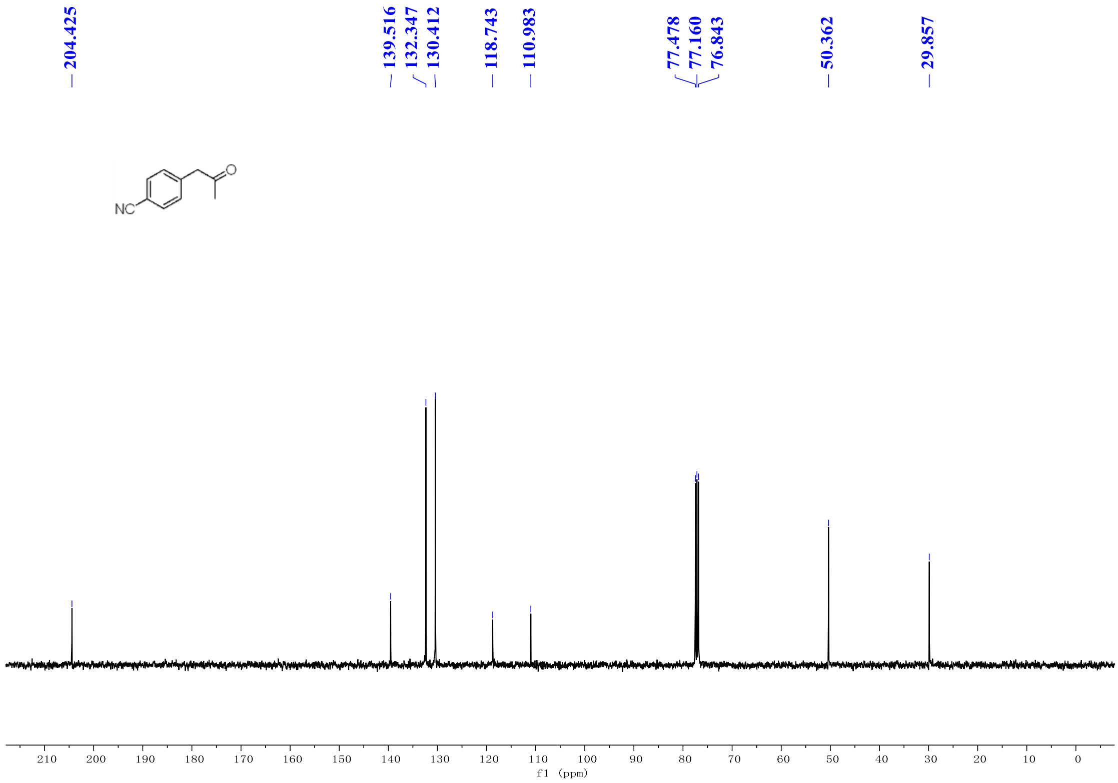

^{1}H NMR spectrum (400 MHz, $CDCl_3$) of 1-(4-nitrophenyl)propan-2-one (**4j**)

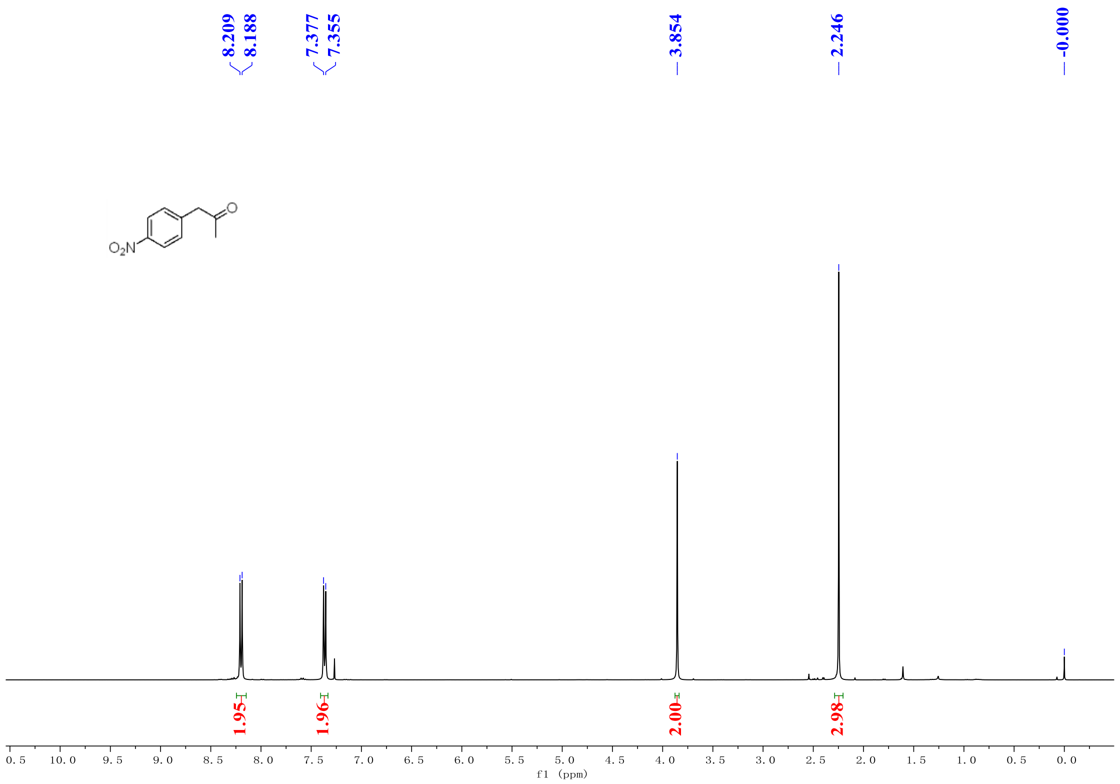

^{13}C NMR spectrum (101 MHz, $CDCl_3$) of 1-(4-nitrophenyl)propan-2-one (**4j**)

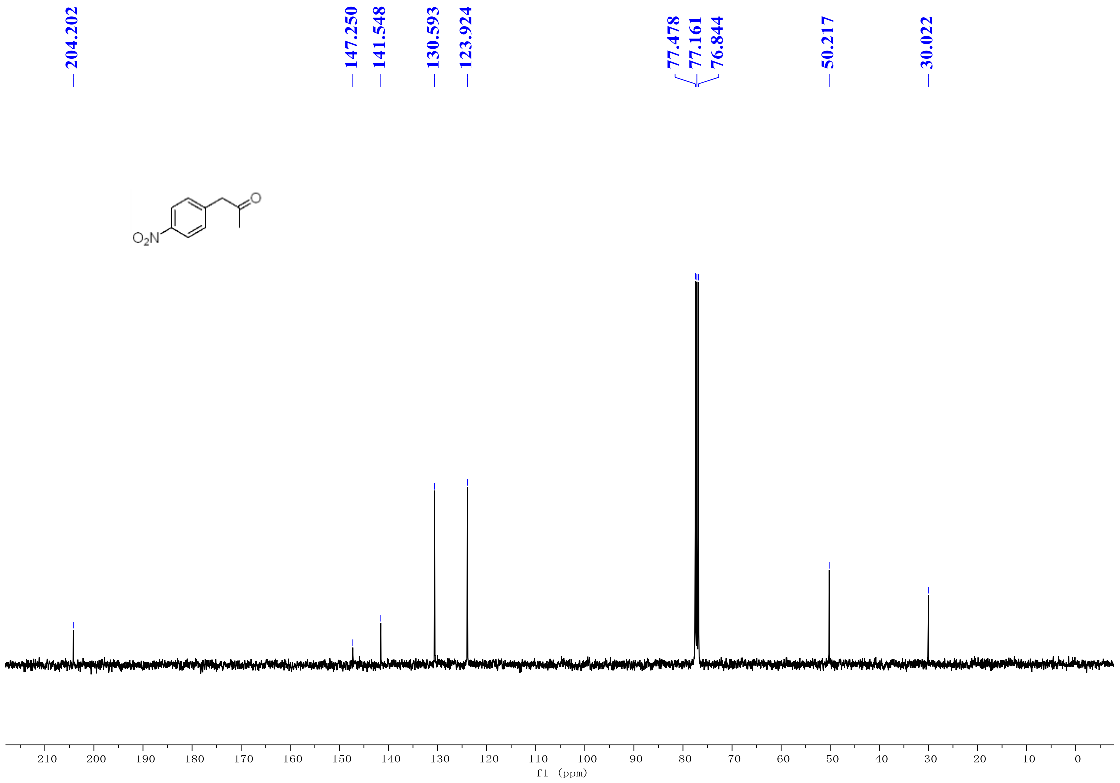

^{1}H NMR spectrum (400 MHz, $CDCl_3$) of 1-(4-(methylsulfonyl)phenyl)propan-2-one **(4k)**

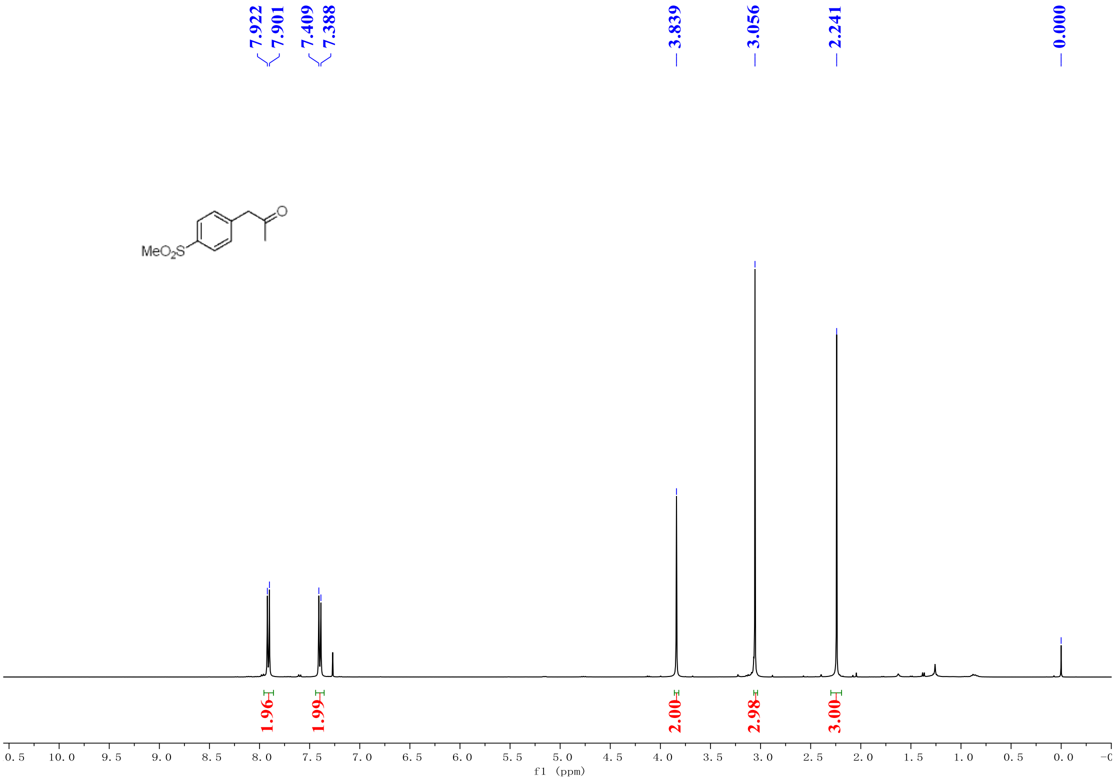

^{13}C NMR spectrum (101 MHz, $CDCl_3$) of 1-(4-(methylsulfonyl)phenyl)propan-2-one **(4k)**

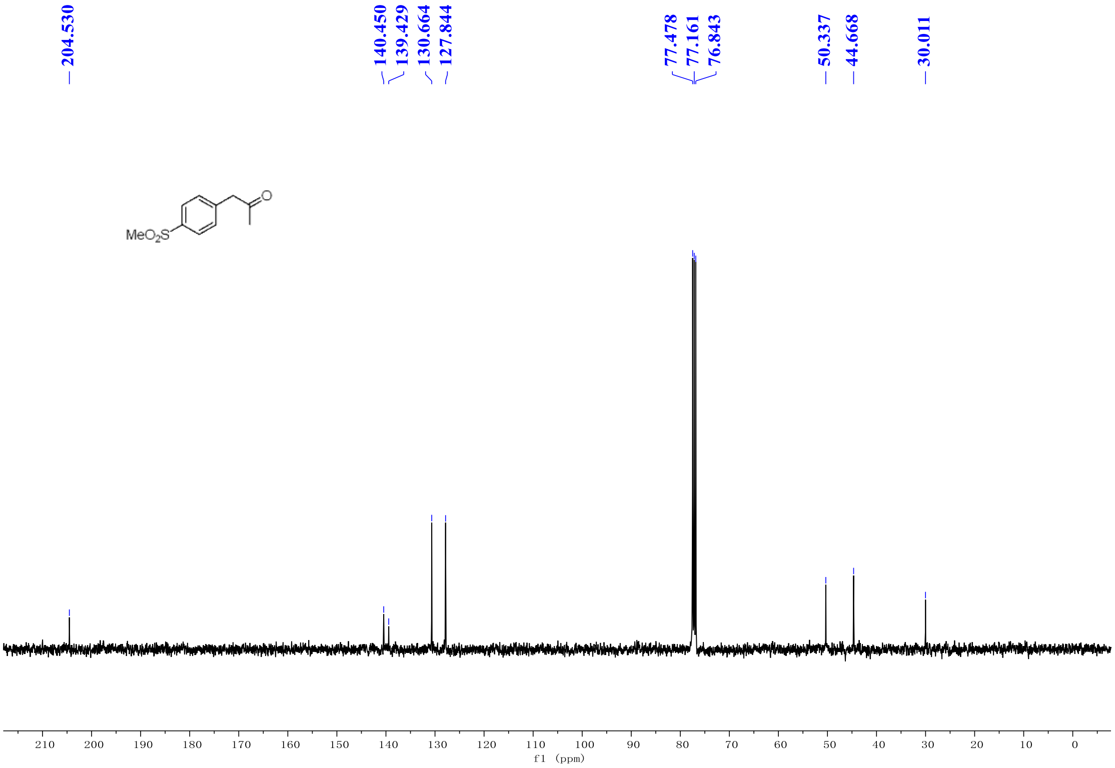

^{1}H NMR spectrum (400 MHz, $CDCl_3$) of 1-phenylbutan-2-one (**4l**)

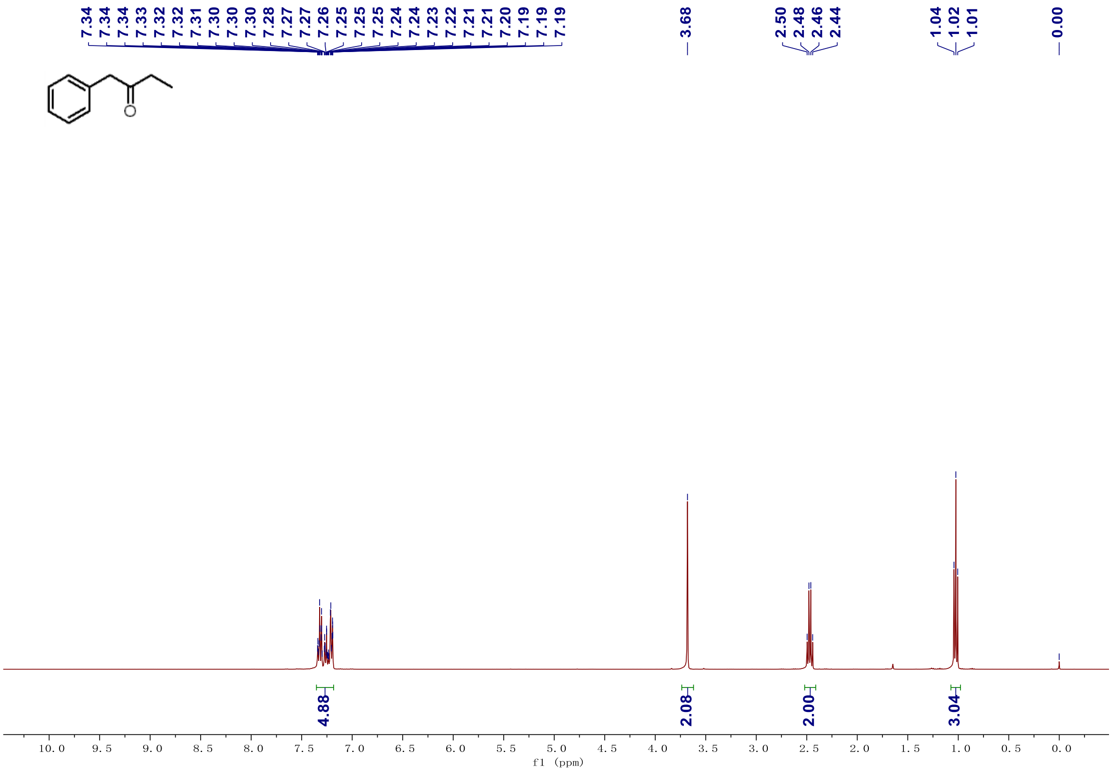

^{13}C NMR spectrum (101 MHz, $CDCl_3$) of 1-phenylbutan-2-one (**4l**)

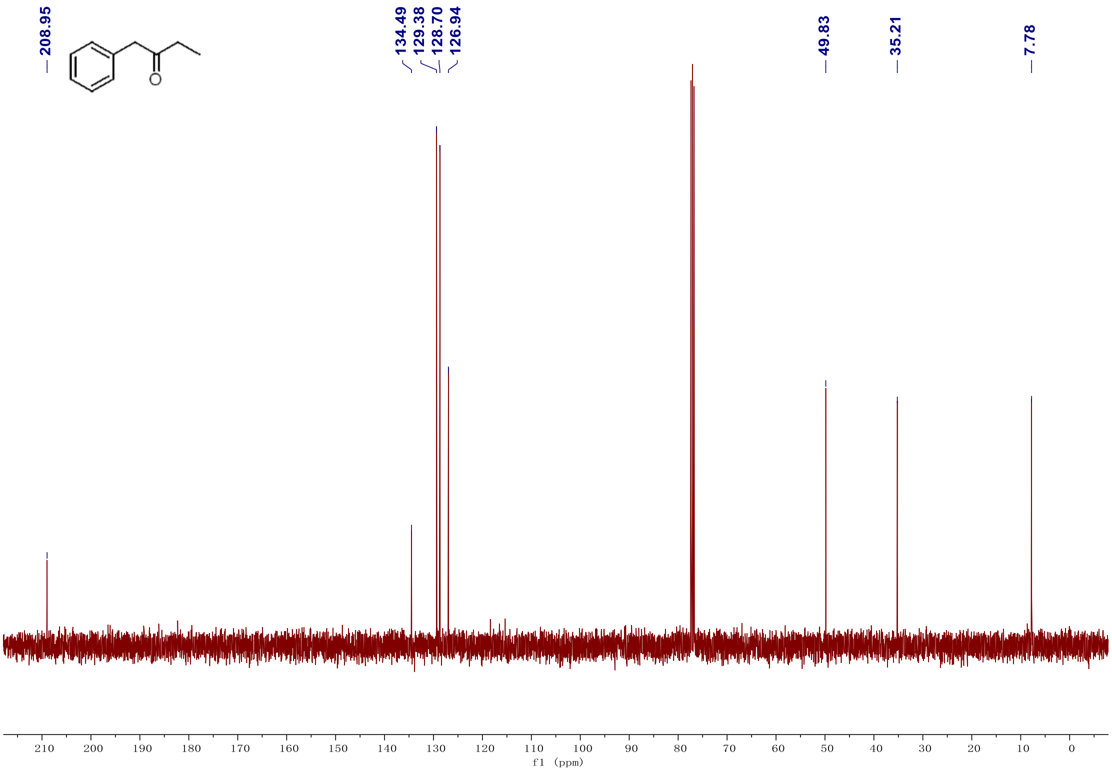

3.2 ¹H NMR for Reaction Mixtures in Table 1

Table 1, entry 1

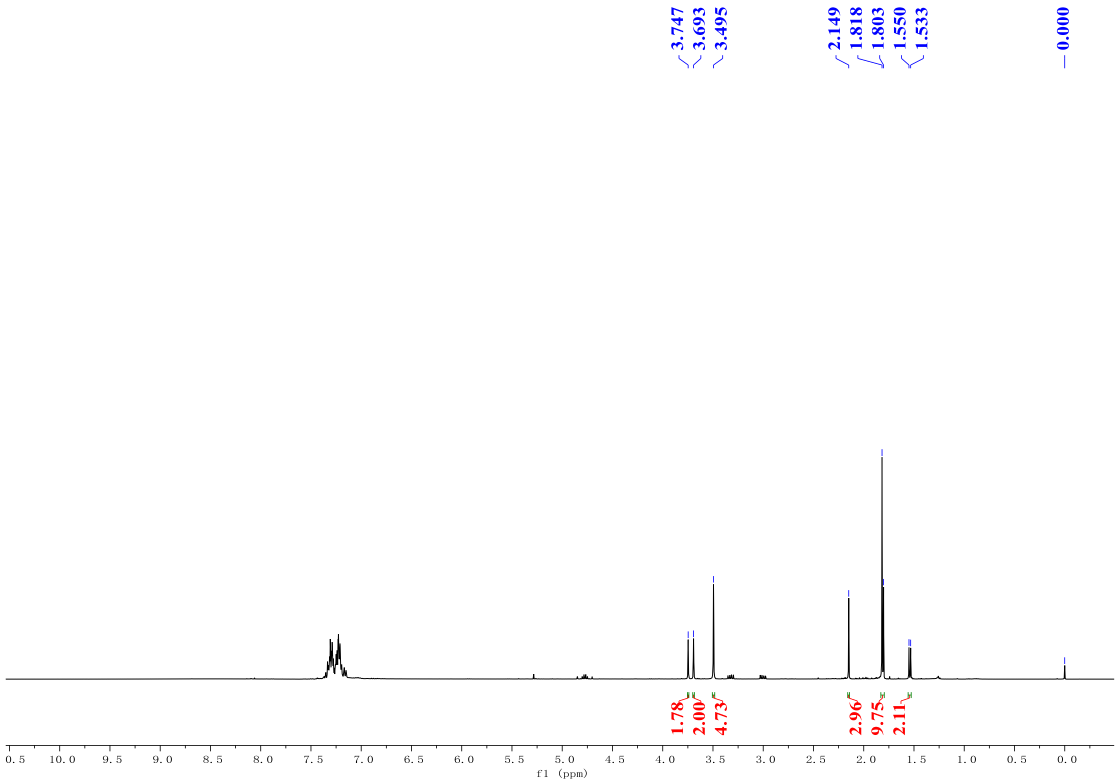

Table 1, entry 2

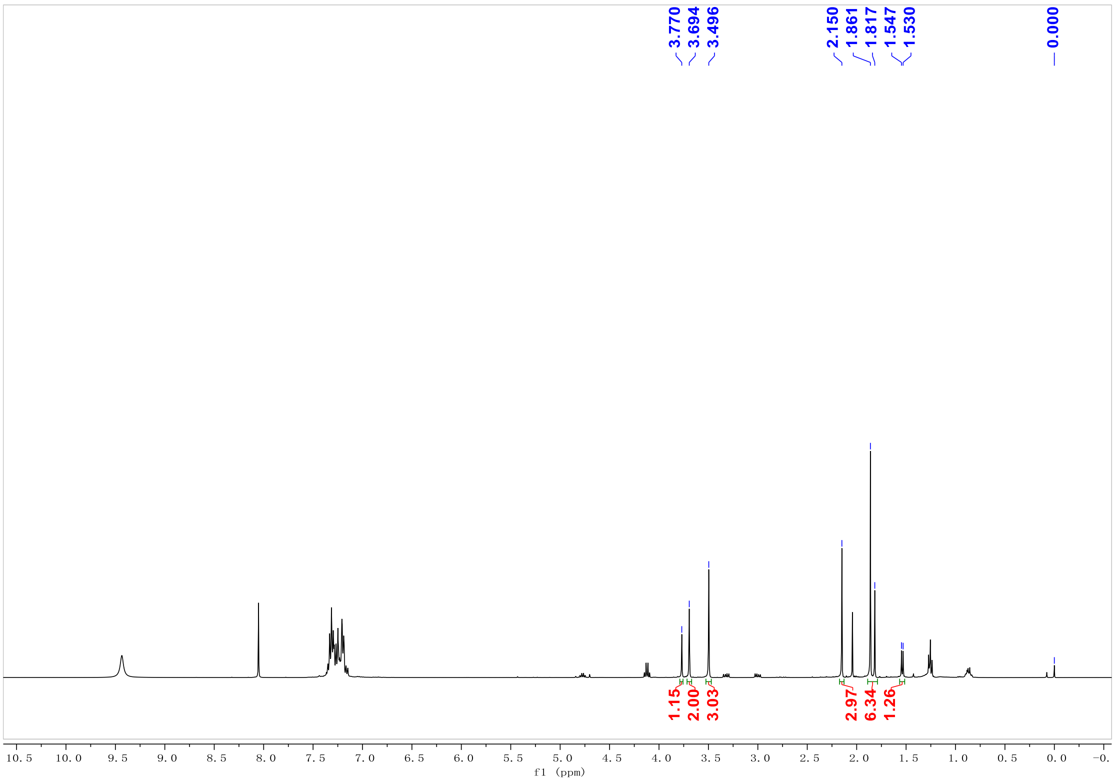

Table 1, entry 3

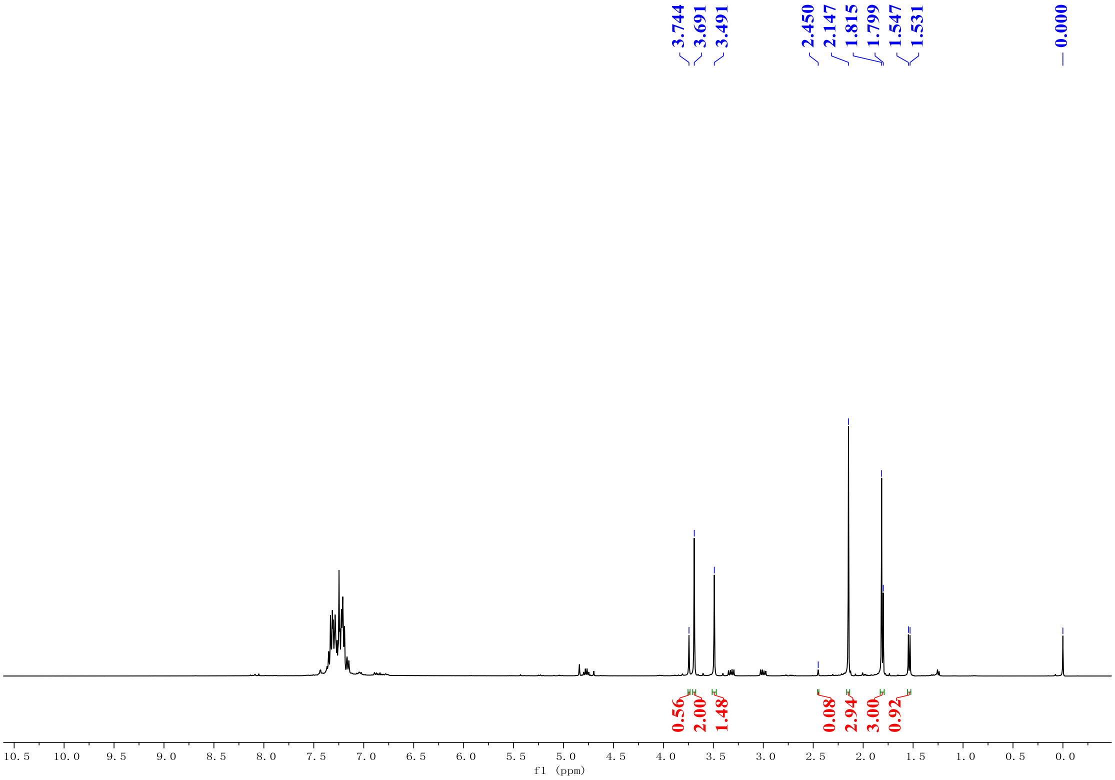

Table 1, entry 4

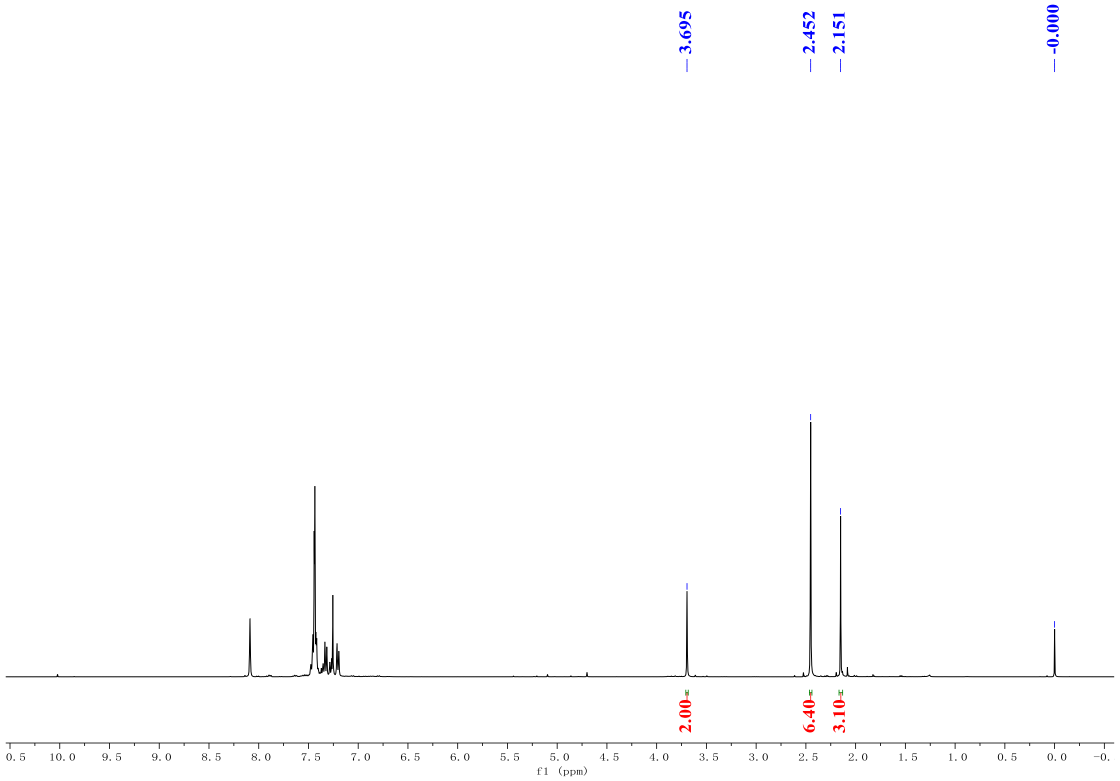

Table 1, entry 5

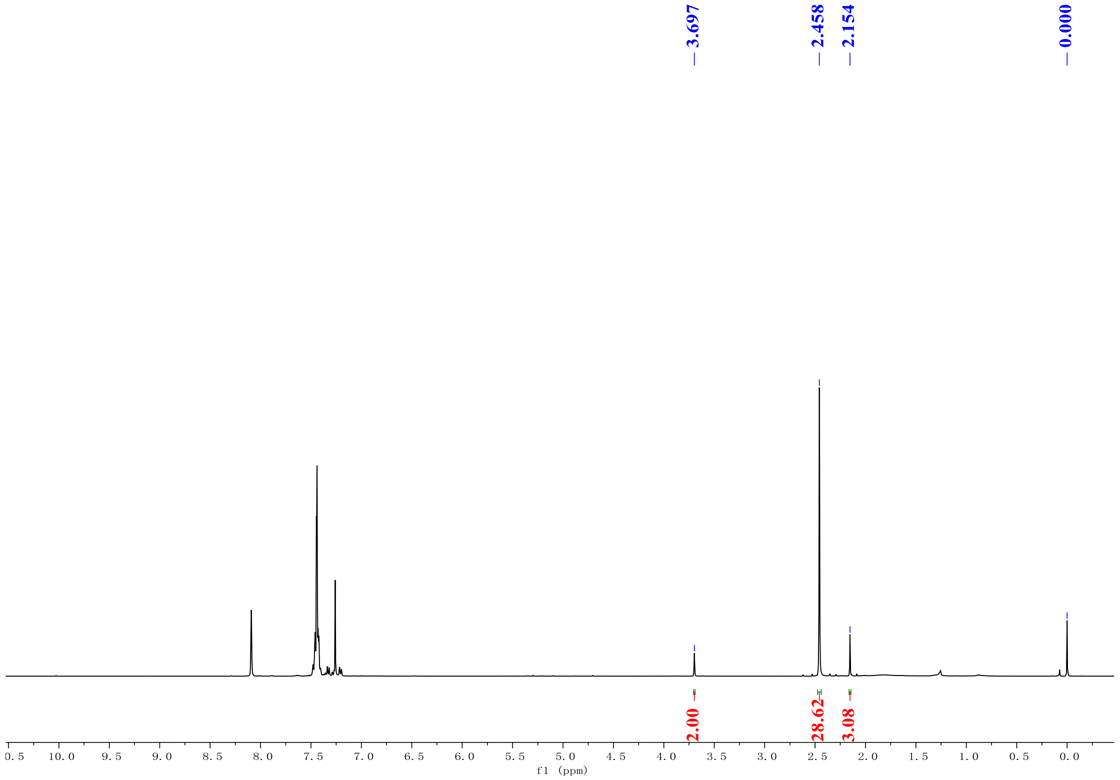

Table 1, entry 6

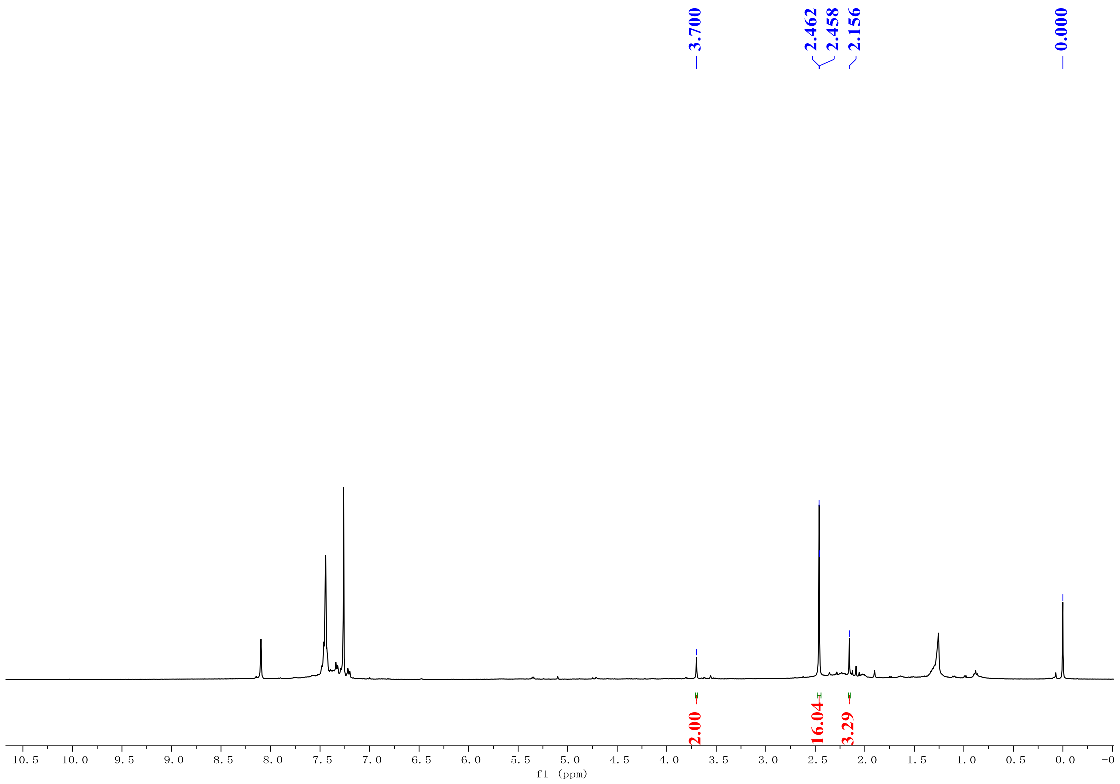

Table 1, entry 7

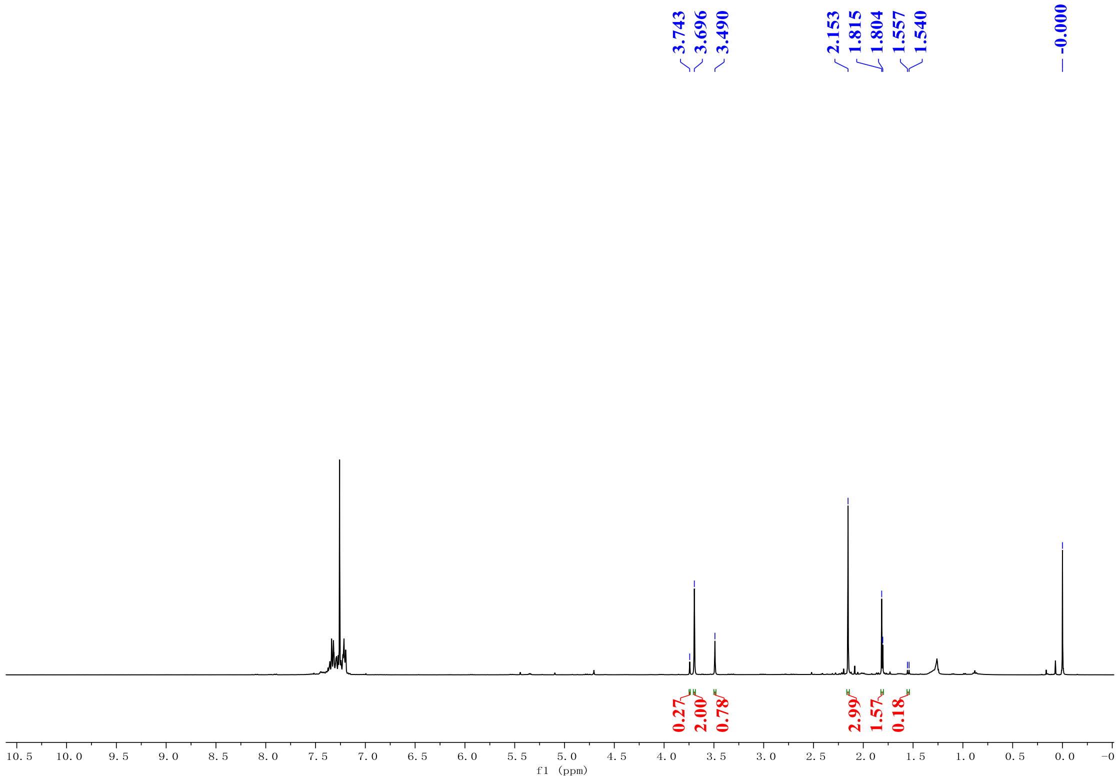

Table 1, entry 8

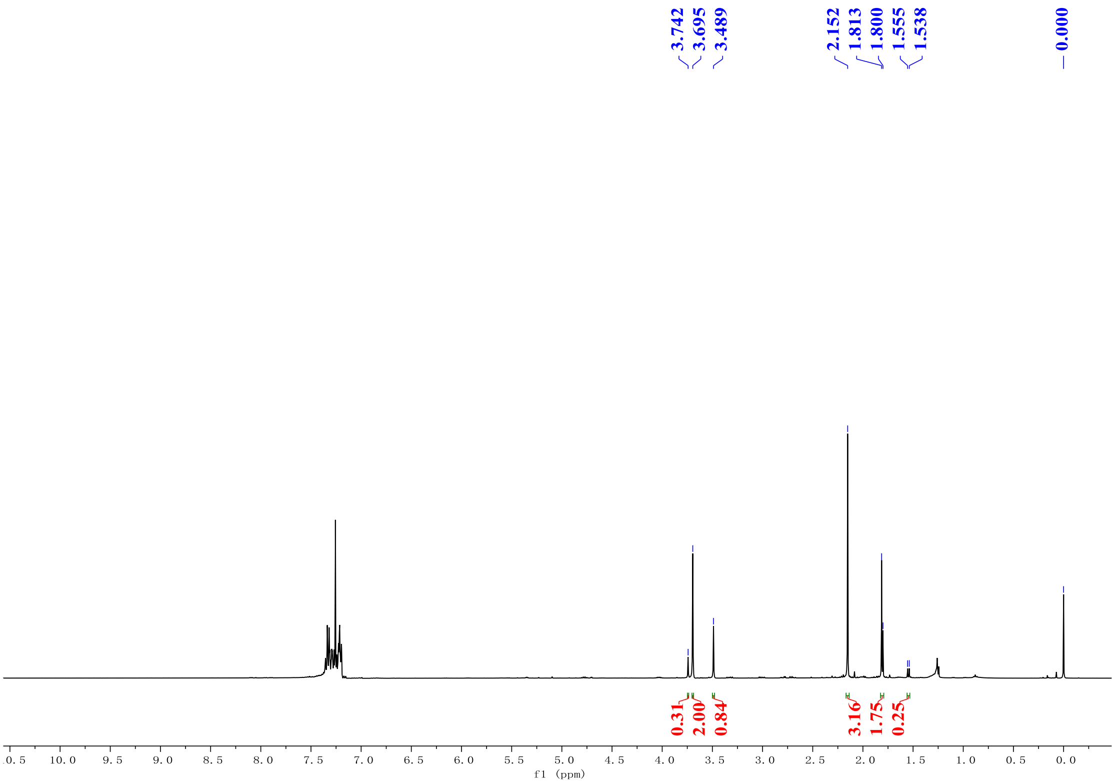

Table 1, entry 9

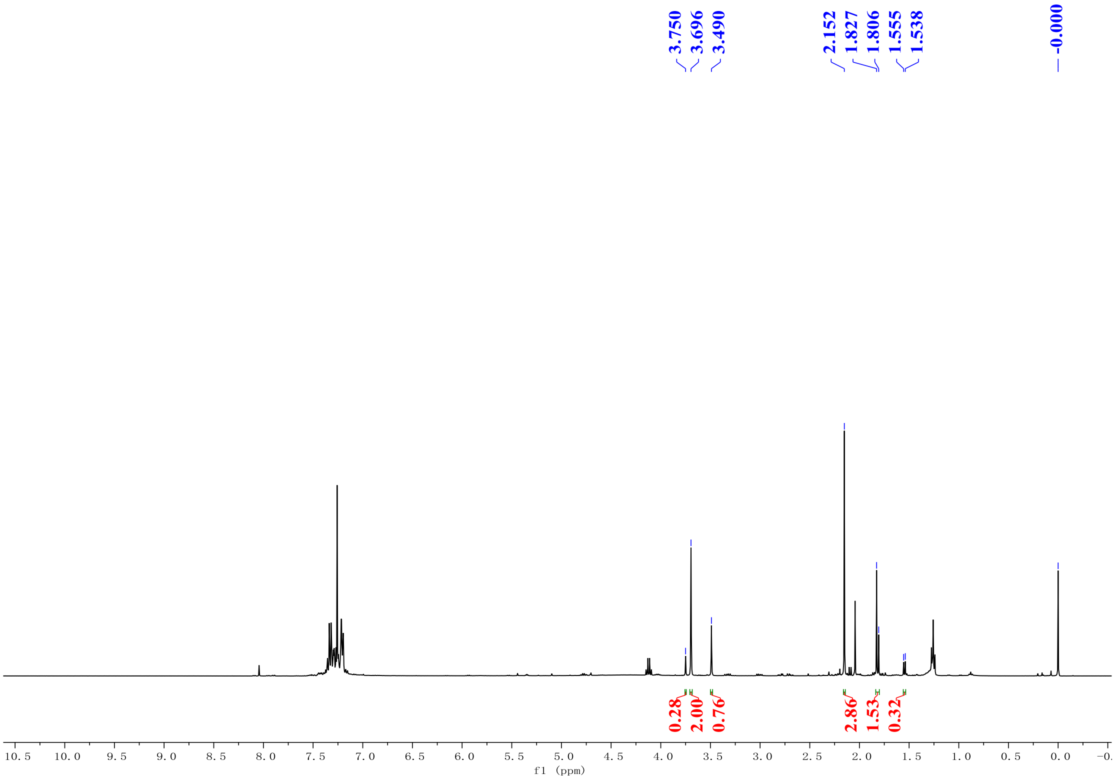

Table 1, entry 10

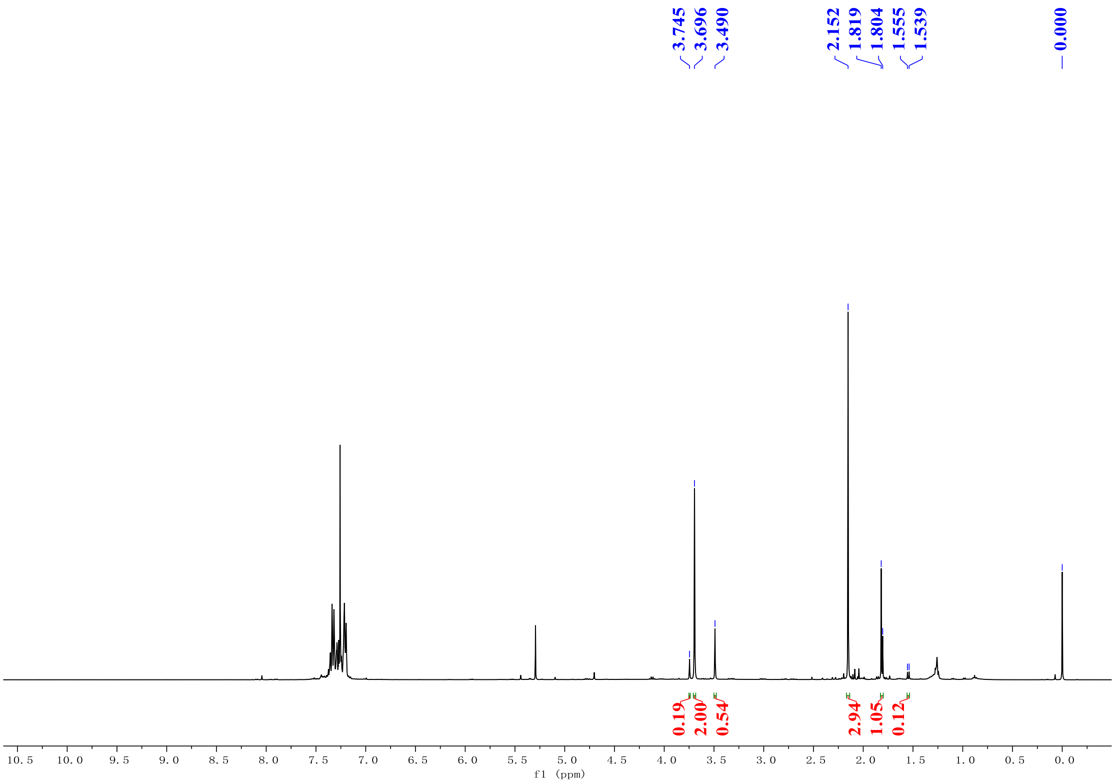

Table 1, entry 11

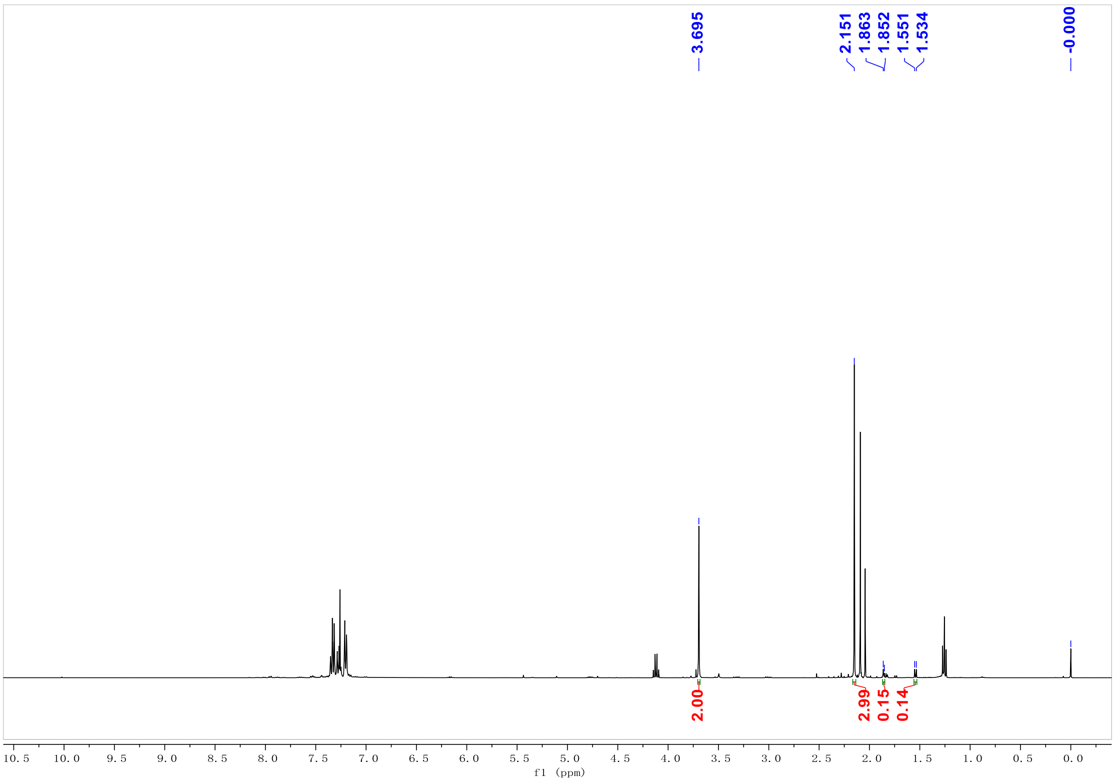

Table 1, entry 12

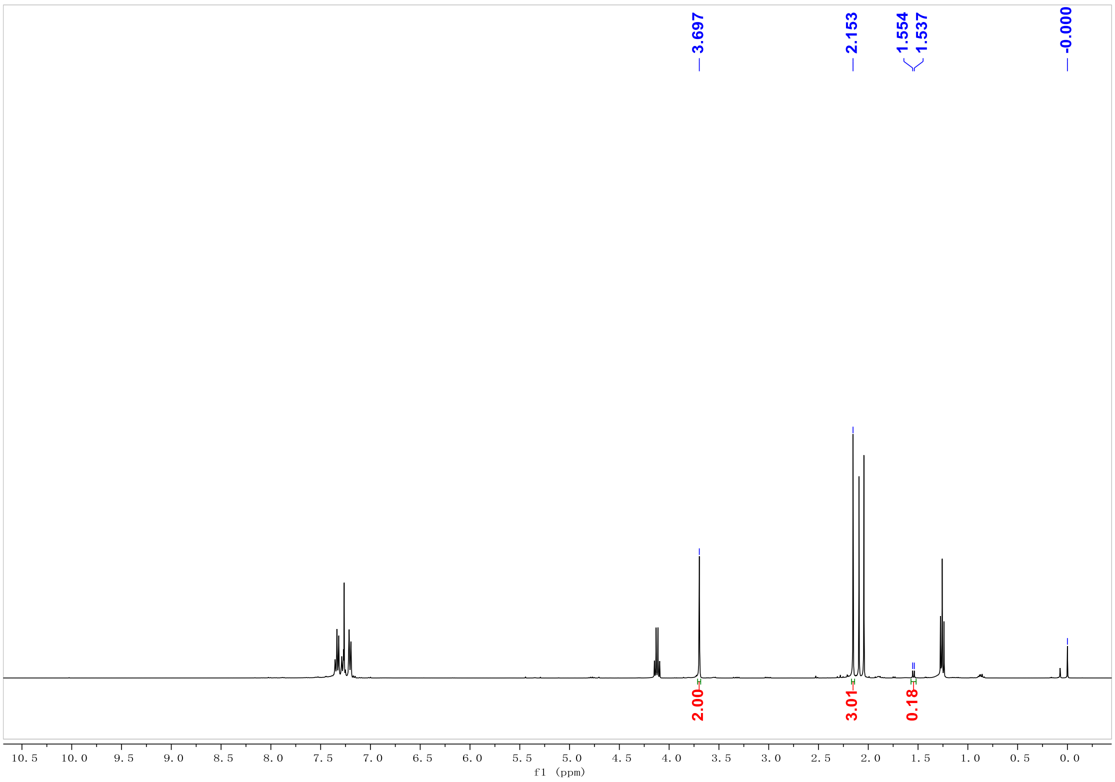

Table 1, entry 13

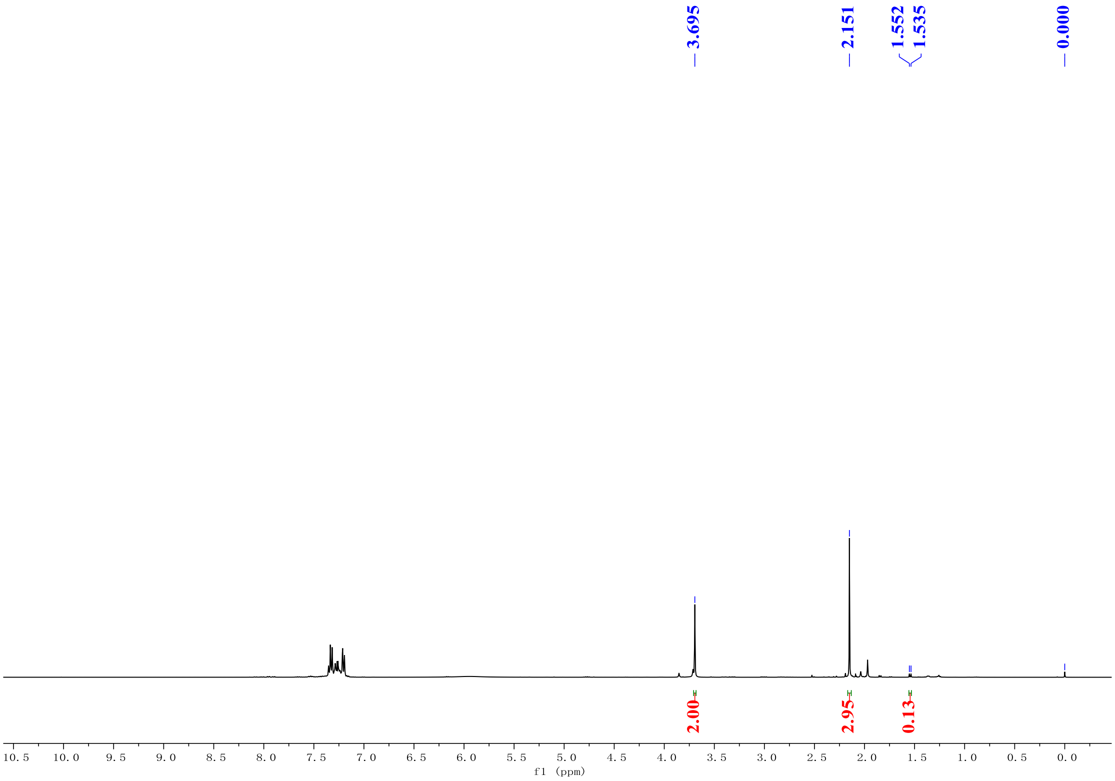

Table 1, entry 14

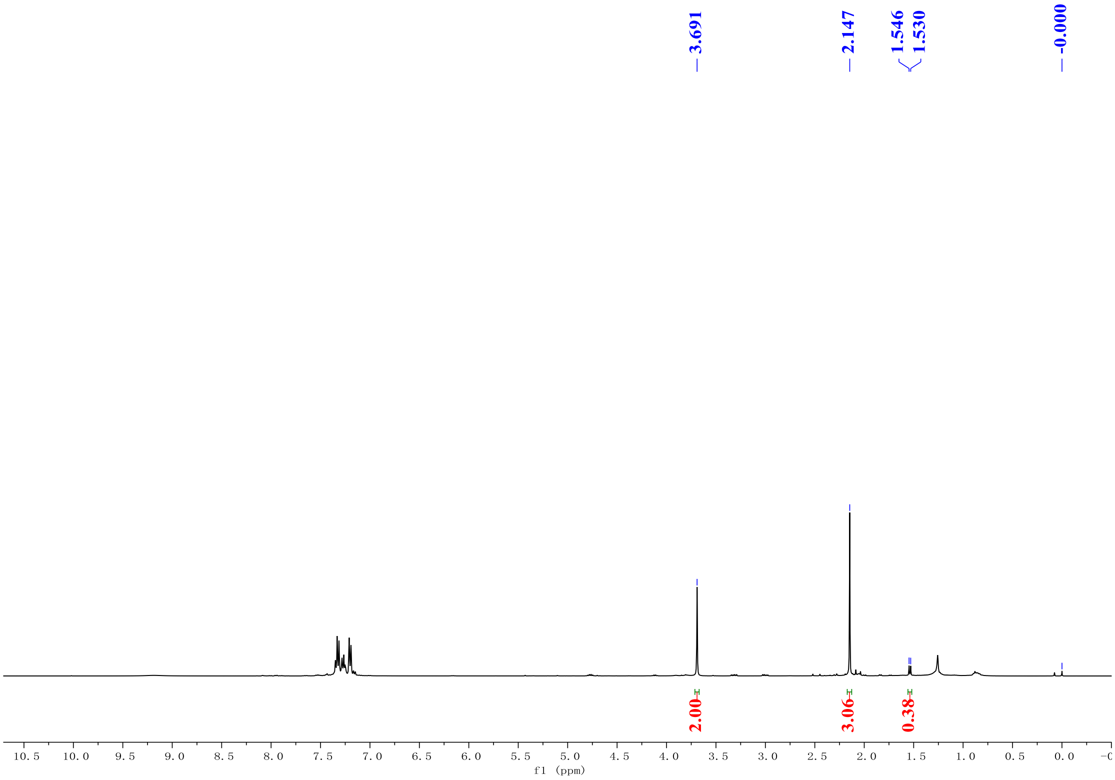

Table 1, entry 15

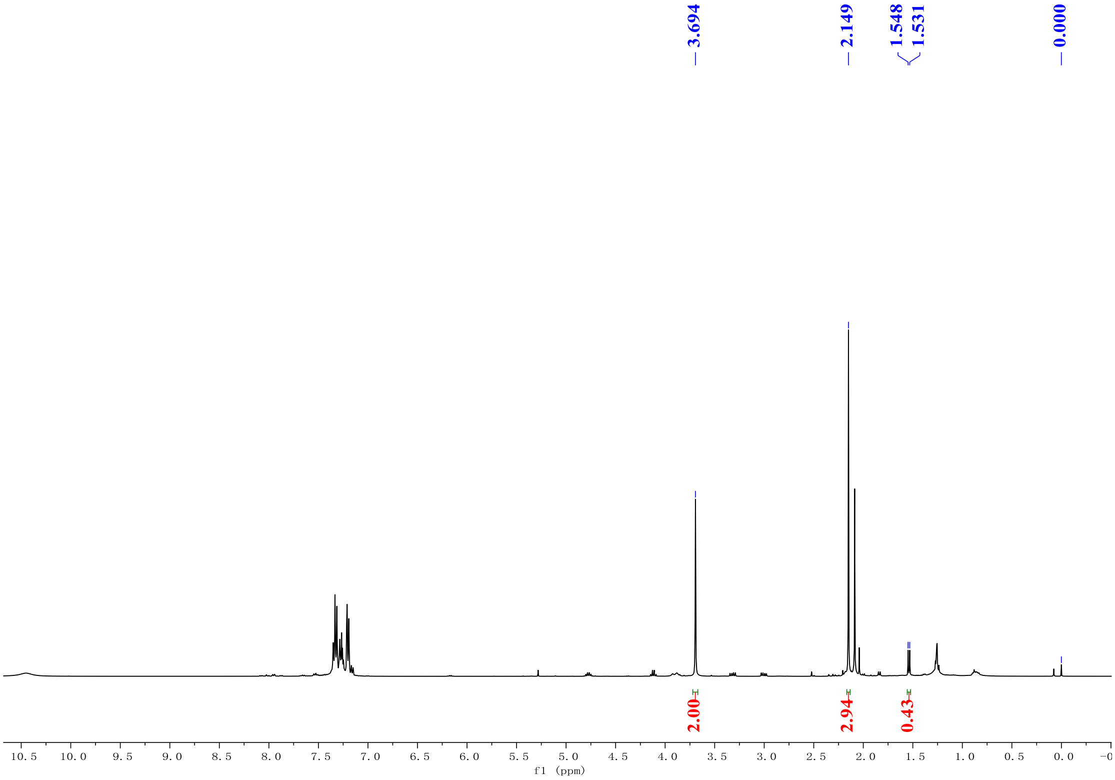

Table 1, entry 16

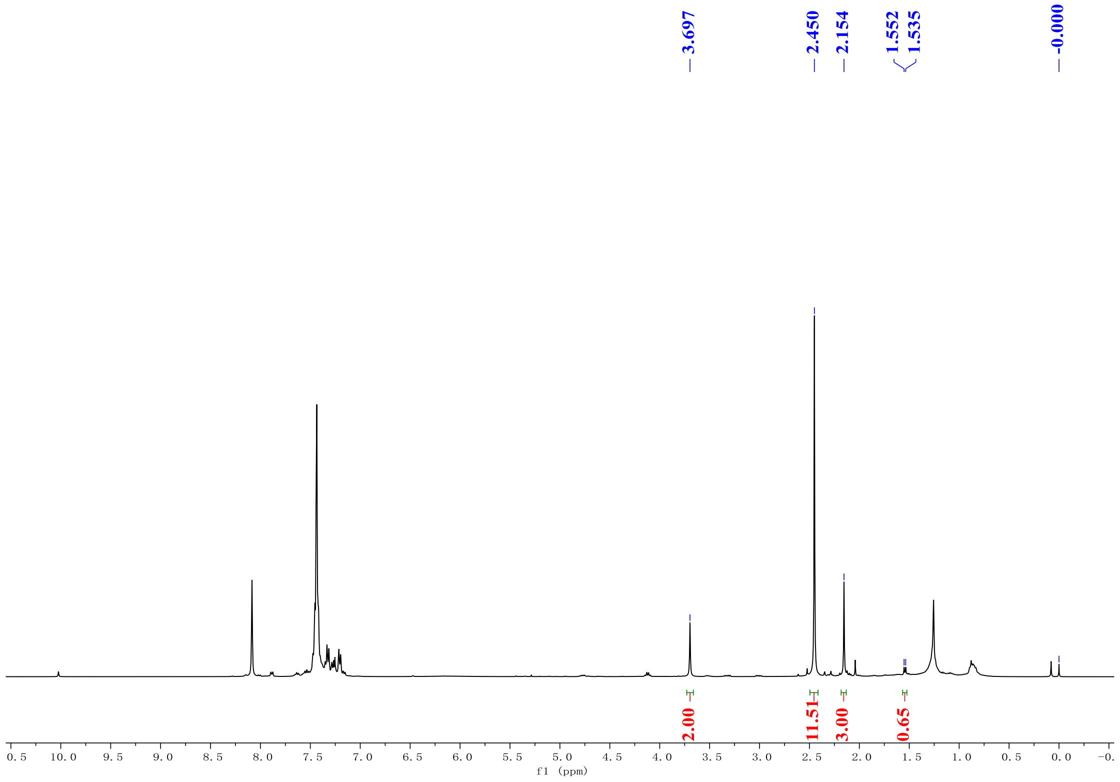

Table 1, entry 17

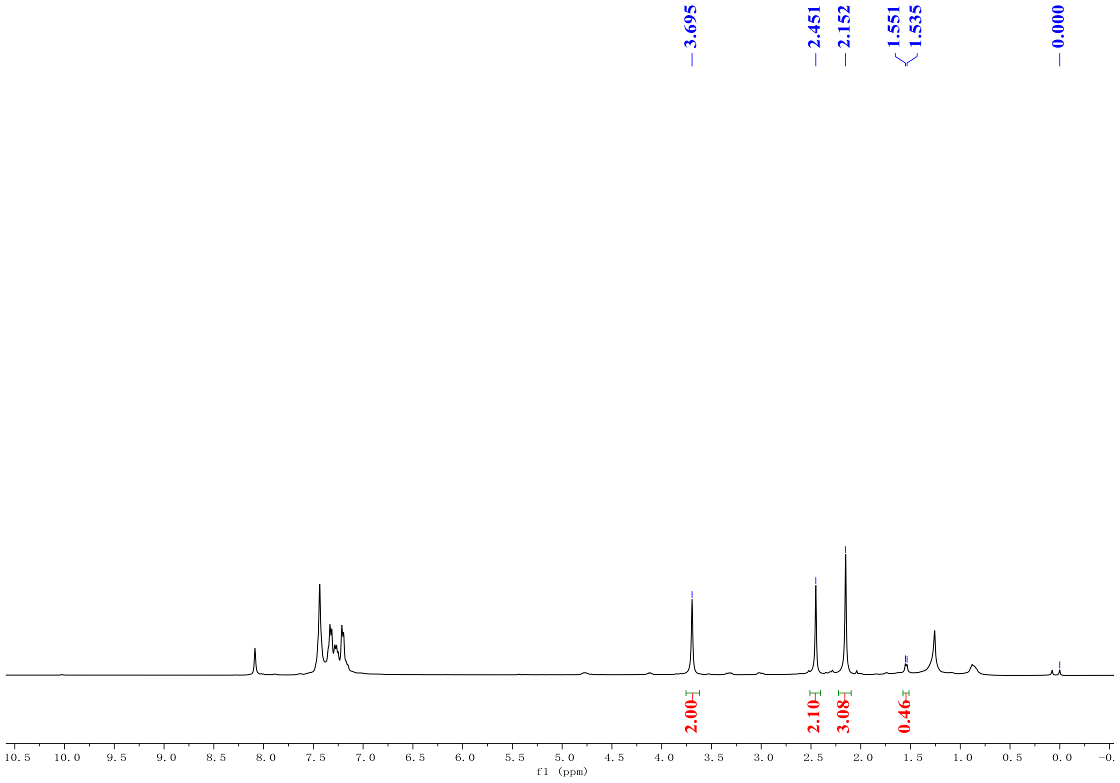

Table 1, entry 18

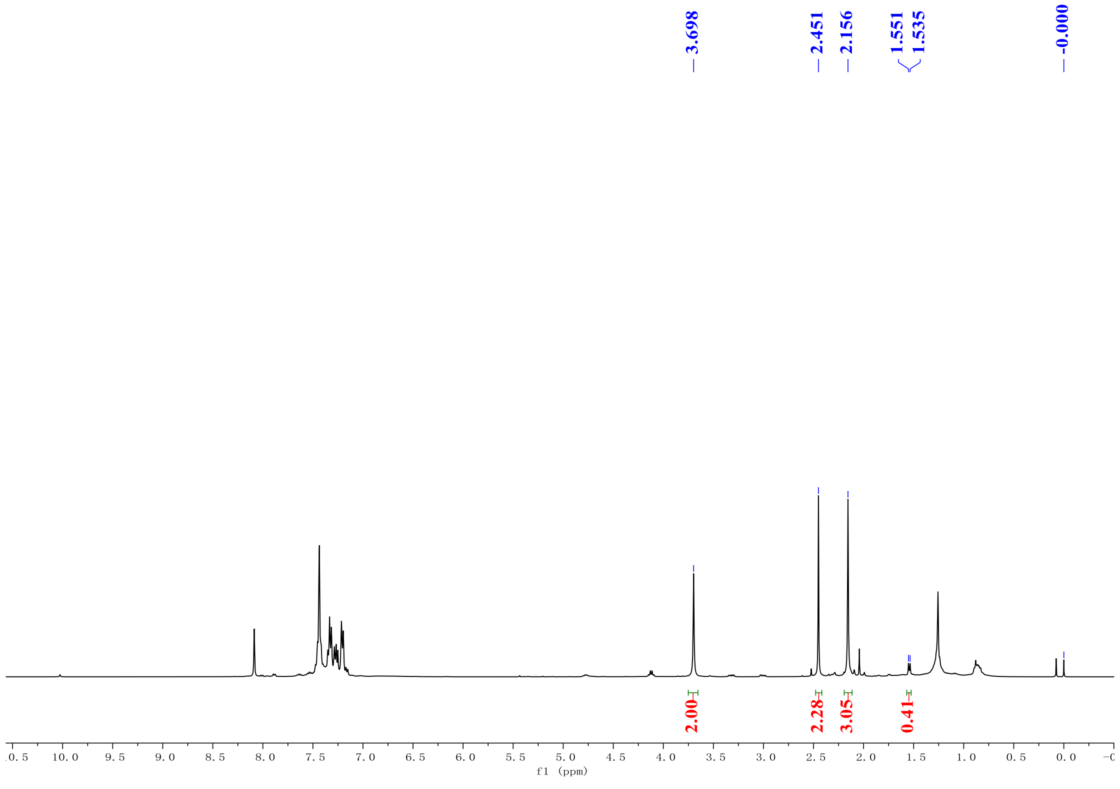

Table 1, entry 19

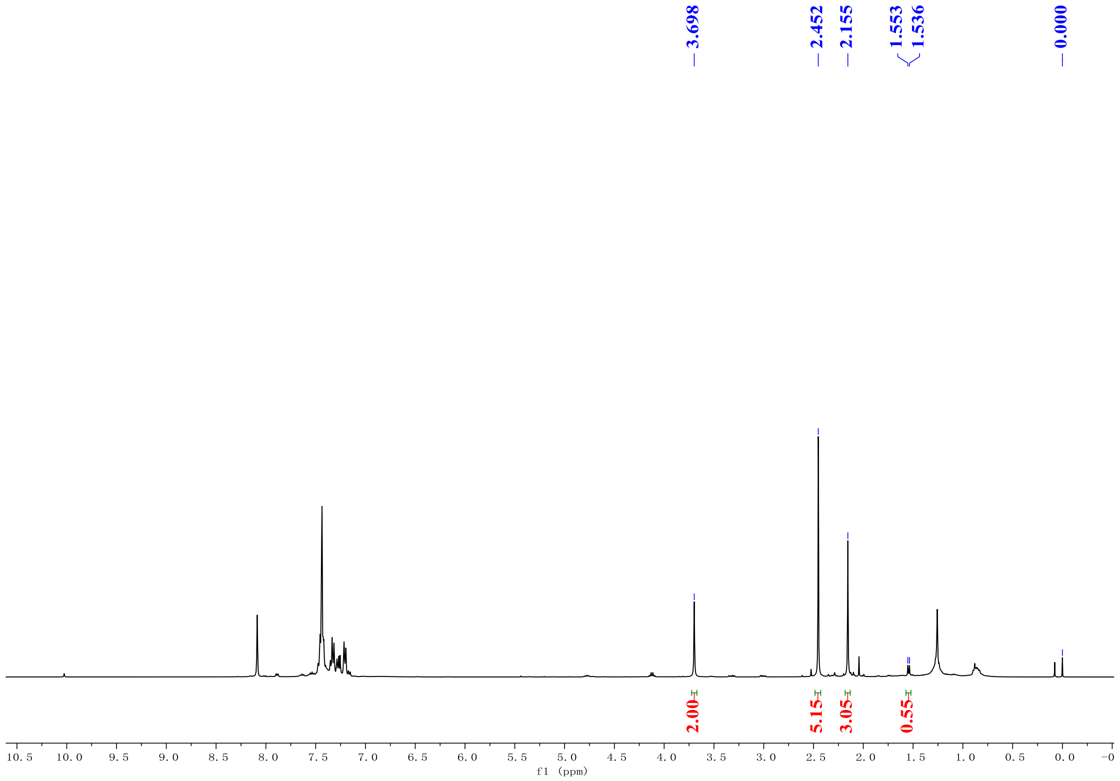

Table 1, entry 20

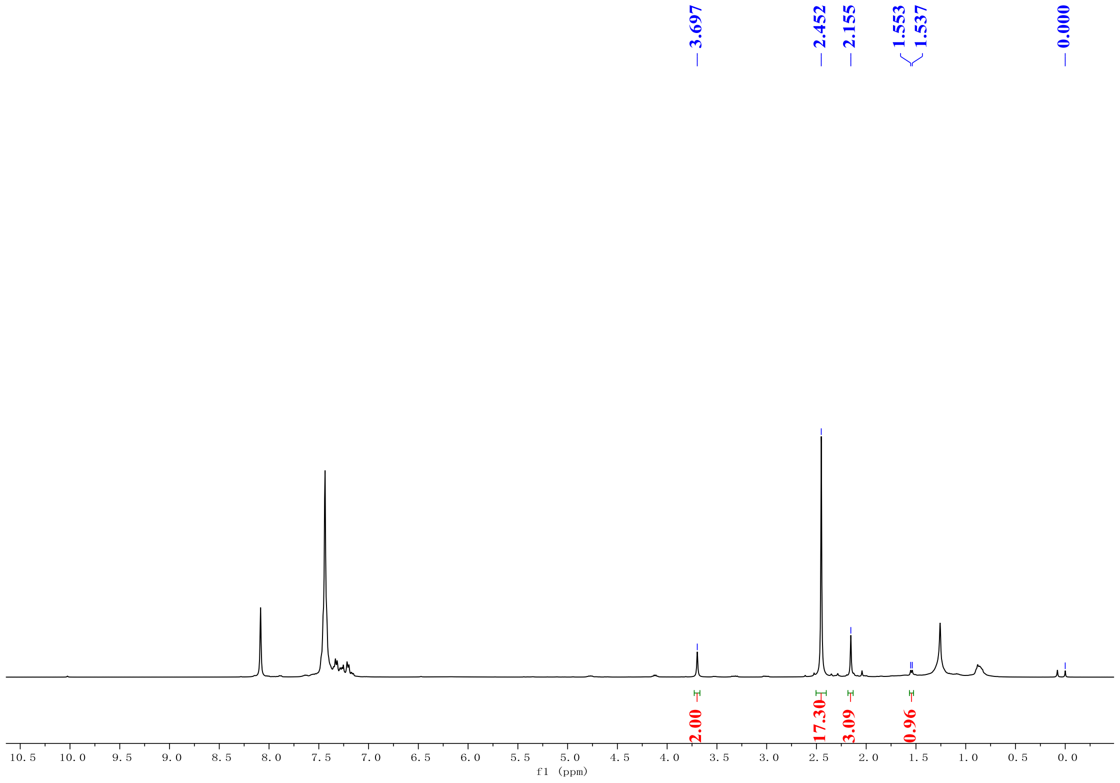

Table 1, entry 21

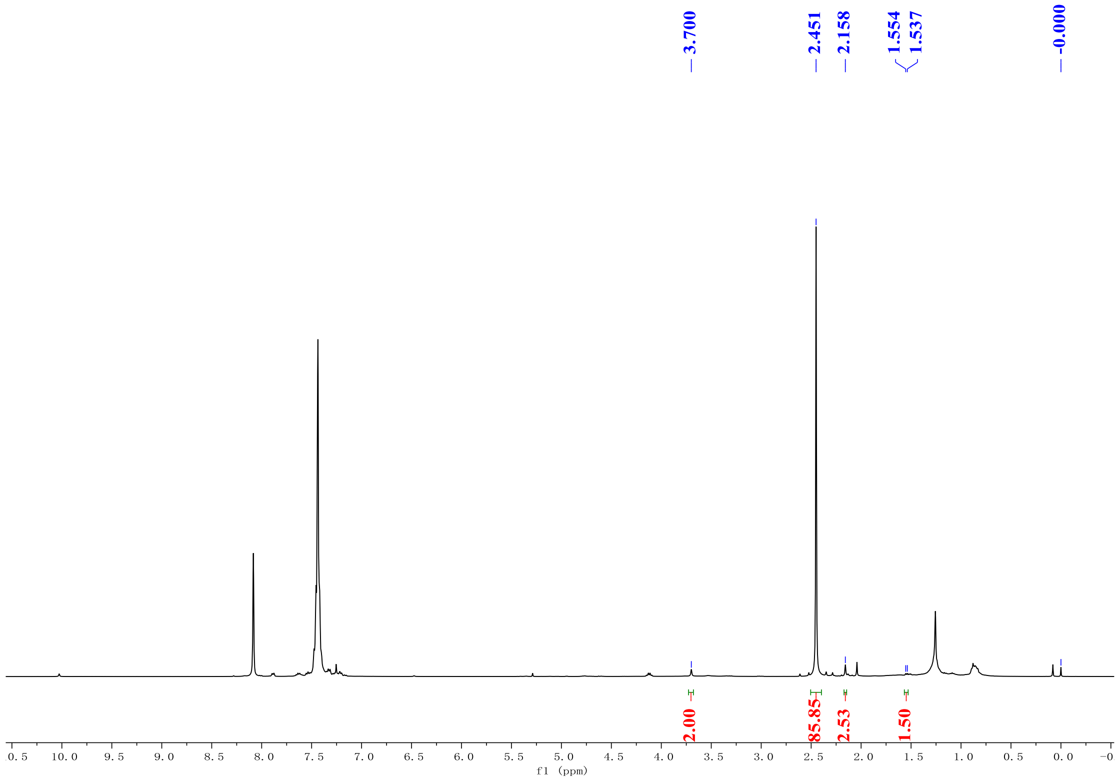

Supplement: Supplementary file 1 [file molecules-27-07822-s001.zip › molecules-2024023-supplementary.pdf]
